# Phenotypes of Myopathy-Related Beta-Tropomyosin Mutants in Human and Mouse Tissue Cultures

**DOI:** 10.1371/journal.pone.0072396

**Published:** 2013-09-10

**Authors:** Saba Abdul-Hussein, Karin Rahl, Ali-Reza Moslemi, Homa Tajsharghi

**Affiliations:** 1 Department of Pathology, University of Gothenburg, Gothenburg, Sweden; 2 Department of Clinical and Medical Genetics, University of Gothenburg, Gothenburg, Sweden; Instituto de Ciencia de Materiales de Madrid – Instituto de Biomedicina de Valencia, Spain

## Abstract

Mutations in *TPM2* result in a variety of myopathies characterised by variable clinical and morphological features. We used human and mouse cultured cells to study the effects of β-TM mutants. The mutants induced a range of phenotypes in human myoblasts, which generally changed upon differentiation to myotubes. Human myotubes transfected with the E41K-β-TM_EGFP_ mutant showed perinuclear aggregates. The G53ins-β-TM_EGFP_ mutant tended to accumulate in myoblasts but was incorporated into filamentous structures of myotubes. The K49del-β-TM_EGFP_ and E122K-β-TM_EGFP_ mutants induced the formation of rod-like structures in human cells. The N202K-β-TM_EGFP_ mutant failed to integrate into thin filaments and formed accumulations in myotubes. The accumulation of mutant β-TM_EGFP_ in the perinuclear and peripheral areas of the cells was the striking feature in C2C12. We demonstrated that human tissue culture is a suitable system for studying the early stages of altered myofibrilogenesis and morphological changes linked to myopathy-related β-TM mutants. In addition, the histopathological phenotype associated with expression of the various mutant proteins depends on the cell type and varies with the maturation of the muscle cell. Further, the phenotype is a combinatorial effect of the specific amino acid change and the temporal expression of the mutant protein.

## Introduction

Tropomyosin (TM) is a component of the muscle sarcomeric thin filament where it plays a central role in the calcium-dependent regulation of striated muscle contraction. TM exists as a rod-shaped dimer that forms a head-to-tail polymer along the length of an actin filament providing stability and it is essential for myosin-actin interaction [1], [2]. In tropomyosin a heptad repeat motif forms a parallel α-helical coiled-coil structure, which is required for the correct formation of dimers, as well as for the interaction with proteins along the length of actin thin filaments [2]–[4]. TMs comprise a family of proteins encoded by four different genes (*TPM1*, *TPM2*, *TPM3* and *TPM4*) [2]. In human striated muscle, there are three major tropomyosin isoforms: α-TM, β-TM and γ-TM, encoded by the *TPM1*, *TPM2* and *TPM3* genes respectively. Beta-tropomyosin (β-TM) is mainly expressed in slow, type 1 and to some extent in fast muscle fibres and cardiac muscle [5]. The α-TM muscle isoform is predominantly expressed in cardiac muscle and fast type 2, muscle fibres, whereas γ-TM is predominantly expressed in slow muscle fibres but also in the heart [2]. Each gene uses alternative promoters, alternative splicing and differential RNA processing to introduce multiple striated muscle, smooth muscle and cytoskeletal transcripts [2], [6].

The majority of identified mutations in *TPM2* are dominant and occur *de novo* in sporadic cases [7]. They give rise to clinically and morphologically different phenotypes, such as cap disease [8]–[11], nemaline myopathy [8], non-specific congenital myopathies [7], [12], congenital fibre type disproportion [13] and distal arthrogryposis syndromes (DA) type 1 [14] and 2B [15]. In addition, Escobar syndrome with nemaline myopathy has been associated with a homozygous stop mutation in *TPM2*, resulting in the complete absence of β-TM [16]. Nemaline myopathy and cap disease are a subgroup of congenital myopathies and are characterised by the presence of nemaline rods and cap-like structures respectively in muscle biopsy specimens [17], [18]. The nemaline rods are deposits of Z-disk and thin filament material, largely composed of α-actinin and actin [19]. The peripherally located cap-like structures consist of abnormal accumulations of sarcomeric multiprotein inclusions including actin, tropomyosin, desmin, troponin T and nebulin with enlarged Z-disks [18], [20]. The presence of overlapping clinical phenotypes and the changes in the morphological features indicate that cap disease can be regarded as a variant or early stage of nemaline myopathy [8].

The critical regulatory role of TM in muscle contraction, in addition to its function in the maintenance of stable sarcomeric thin filament structures, suggests that a number of pathogenic mechanisms are likely to be associated with mutations in *TPM2*. This postulation has been supported by different *in vivo* and *in vitro* physiological studies that have highlighted functional deficits in the contraction regulation and protein interaction of *TPM2* [21]–[23]. In this case, we have used transfection studies in human and mouse cultured myoblasts and myotubes to characterise the histological behaviour of five β-TM mutants associated with different morphological features. E41K mutation is associated with a congenital myopathy characterised by either cap structures or an accumulation of nemaline rods [8]. K49del, G53ins and N202K mutations are associated with the appearance of cap structures and an abnormal coarse-meshed intermyofibrillar network in the muscle of the patients [10]. E122K mutation is linked to a non-specific congenital myopathy and type 1 fibre predominance [7].

In addition, it has been reported that p62 protein, also known as sequestosome 1 (p62/SQSTM1), functions in aggregate formation and proteolysis [24] [25]. It acts as a shuttle protein transporting polyubiquitinated proteins for degradation by both the proteasome and the lysosome [26], [27]. It has also been indicated that P62 is a component of many disease-associated intracellular ubiquitinated multiprotein aggregates [28], [29]. On this basis, we used immunofluorescence labeling to examine the expression of p62 in transfected cells and study the role of p62 in aggregate formation in our disease models.

## Materials and Methods

### Construction of the enhanced green fluorescent protein (EGFP)-tagged β-tropomyosin mutants

The wild-type skeletal muscle specific *TPM2* cDNA fragment was generated through the amplification of β-tropomyosin from reverse transcribed human skeletal muscle RNA (transcript GI: 47519592, protein accession: P07951.1) using specific primers introducing *Xho*I (5′) and *Eco*RI (3′) restriction sites. The polymerase chain reaction (PCR) product was cloned into a PCRII-Blunt-TOPO-vector (Invitrogen, Carlsbad, CA, USA), according to the manufacturer's instructions. The cloned PCR product was cut with *Xho*I and *Eco*RI restriction enzymes and gel purified using a QIAquick Gel extraction kit (Qiagen, Hilden, Germany). The gel-purified *TPM2* fragment with *Xho*I (5′) and *Eco*RI (3′) restriction sites was subcloned into the *Xho*I and *Eco*RI restriction sites of the pEGFP-N1 to generate WT-TPM2_EGFP_. All *TPM2* mutant constructs were generated in the pEGFP-N1 backbone (Clontech, USA). EGFP-tagged β-tropomyosin mutants (E41K, K49del, G53ins, E122K, N202K) were generated through site-directed mutagenesis and cloning into *E. coli* using a QuickChange^w^Site-Directed Mutagenesis kit (Stratagene, UK), as previously described [30]. WT- and mutant-TPM2_EGFP_ constructs were sequenced to verify the complete *TPM2* coding sequence and correct introduction of the desired mutations.

Wild-type and mutants E41K-, K49del- and G53ins β-TM cDNAs were excised from the β-TM-EGFP constructs, with *Xho*I (5′) and *Eco*RI (3′) restriction sites, and subcloned into the same sites of pcDNA3.1 to generate untagged constructs. In the vectors pEGFP-N1 and pcDNA3.1 the WT- and mutant-TPM2 genes are under control of the human Cytomegalovirus (CMV) promoter.

### Culture of myoblasts

The standardised human myoblast batches were provided by MYOSIX through a collaborative programme with Association Française contre les Myopathies (AFM). The skeletal muscle cells from a donor with no clinical signs of muscle disease were enzymatically isolated and cultured, as previously described [31], [32]. Mouse C2C12 myoblasts were obtained from American Type Culture Collection (ATCC) (LGC Standards AB, Sweden). All reagents were purchased from Invitrogen Life Technologies (Invitrogen, Carlsbad, CA, USA), unless otherwise specified. The human myoblasts in their second passage and C2C12 myoblasts were cultured at 37°C in a humidified 5% CO_2_ atmosphere in a proliferation medium composed of Dulbecco's modified Eagle's (DMEM) (Biochrom AG, Berlin, Germany), supplemented with 20% fetal bovine serum. For human myoblasts, proliferation medium was additionally supplemented with 100× GlutaMAX (Invitrogen, GIBCO, Paisley, UK), 10 mg/ml penicillin/streptomycin (Biochrom AG, Berlin, Germany), 10 µg/ml insulin (Biochrom AG, Berlin, Germany) and 10 ng/ml fibroblast growth factor (BD Biosciences, Bedford, MA, USA).

### Transfection

100×10^3^ cells of C2C12 and human myoblasts were plated on chamber slides (Lab-Tek™ II – CC2™, Nalge Nunc International, Naperville, USA) in medium without antibiotics one day prior to transfection. Cells were transfected at 85%–90% confluence using Lipofectamine 2000™ transfection reagent, according to the manufacturer's instructions. Per 2 cm^2^ culture area, 0.6 µg of DNA was diluted in 50 µl of medium without serum and incubated at room temperature for 5 min. The diluted DNA was combined with Lipofectamine at a ratio of 1∶2 for human cells and 1∶1 for mouse cells. The cells were incubated overnight at 37°C in a humidified 5% CO_2_ atmosphere. Cells were fixed and labeled for immunofluorescence (undifferentiated myoblasts). To induce the differentiation of transfected myoblasts, cells were washed in differentiated medium, composed of DMEM supplemented with 5% horse serum, 10 µg/ml insulin and 10 mg/ml penicillin, and further incubated in this medium for three to six days (D3–6 differentiated myotubes). The transfection experiments were conducted in duplicate and repeated at least ten times for each transfected β-TM construct.

### Phalloidin labeling and confocal microscopy

At given time points, cells plated on chamber slides were washed three times in phosphate-buffered saline (PBS) and fixed with 4% formaldehyde for 10 minutes. Free aldehyde groups were blocked with 50mM NH_4_Cl for 10 minutes and cells were permeabilised in PBS containing 0.1% Triton X-100 for 4 minutes. Cells were incubated with Alexa Fluor® 555 Phalloidin for 20 minutes in the dark. Finally, the slides were mounted using Prolong® Gold antifade reagent with DAPI to highlight cell nuclei. The slides were left overnight in the dark at room temperature before examination. Stained cells were imaged using a Zeiss LSM 510 Meta confocal microscope, using 63× oil and 40× objectives, or an LSM 700 inverted Axio Observer.Z1 microscope, using a Plan-Apochromat 63×/1.4 Oil DIC or 40× objective. Images were processed using Photoshop software (Adobe, USA).

### Immunofluorescence analysis of differentiated cells

In order to assess the myofibrils and determine whether they showed signs of maturity the presence of typical sarcomeric Z-disk, A/I-junctions, A-bands and M-bands structures was analysed by immunostaining. Cells were incubated with anti-Z-disk titin (T12) [33], anti-A/I-junction titin (T3) [33], anti-A-band titin (T30) [34] and anti-M-band titin (T51) [35], followed by a secondary Polyclonal Rabbit Anti-Mouse Immunoglobulins/FITC, diluted 1∶1000 (Dako, Sweden AB). All anti-titin antibodies were a gift from Dr. Fürst (Institute of Cell Biology, University of Bonn, Germany).

In addition, immunolabeling of three-day differentiated myotubes with various antibodies against MyHC isoforms was performed to detect different degrees of maturation. Cells were incubated with anti-embryonic-MyHC, diluted 1∶10, anti-fetal-MyHC, diluted 1∶10 and anti-fast IIa+slow-MyHC, diluted 1∶120, followed by a secondary anti-mouse Dylight 549, diluted 1∶1000 (NovoCastra™Lyophilized).

The slides were mounted using Prolong® Gold antifade reagent with blue fluorescent nuclear counterstain DAPI and stained cells were imaged using a Zeiss Axio Observer microscope, using 40× and 63× oil objectives. Images were processed using Photoshop software (Adobe, USA).

### Immunofluorescence analysis of β-TM isoform in human and C2C12 cells

To examine the expression of β-TM isoform in proliferating myoblasts and differentiated human and C2C12 cells, immunofluorescence analysis was performed. Cells were incubated with the primary rabbit polyclonal antibody against β-TM isoform (1∶120), Aviva Systems Biology), followed by a secondary anti-rabbit TexasRed, diluted 1∶250 (Vector Laboratories). The slides were mounted using Prolong® Gold antifade reagent with blue fluorescent nuclear counterstain DAPI and stained cells were imaged using a Zeiss Axio Observer microscope, using a 40× objective.

### Analysis of exogenous and endogenous β-TM isoforms at the protein level in the myoblasts and differentiated myotubes

#### Extraction of whole cultured cell lysates

Cultured transfected myoblasts and myotubes (D6) were washed with cold PBS and collected and homogenized in 100 µl Laemmli sample buffer supplemented with 5% β-mercaptoethanol.

#### Extraction of soluble and insoluble protein pools

For preparation of soluble (cytosolic) and insoluble (myofibrillar) protein pools the cultured transfected myoblasts and myotubes (D6) were washed twice with cold PBS and collected by washing the cells with lysis buffer containing 50 mM morpholinoethanesulfonic acid (MES) pH 6.8, 1 mM EGTA pH 8.0, 50 mM KCL, 1 mM MgCL2, 0.5% Triton-X-100 and protease inhibitor cocktail (1∶500, Sigma). This was followed by centrifugation at 15 000 g for 1 h. The supernatant, representing the cytosolic fraction, was collected and mixed with 4× SDS sample buffer (200 mM Tris-HCL pH 6.8, 200 mM DTT, 8% SDS, 40% glycerol, PI cocktail (1∶125), bromophenol blue (BFB)). The remaining pellet, representing the insoluble fraction, was resuspended in 5× SDS sample buffer and sonicated. Both soluble and insoluble protein fractions were heat inactivated at 94°C for 5 min prior to storage at −20°C.

#### Western blot analysis

After heating to 94°C for two min, extracted protein samples plus 3 M urea were separated by 10% sodium dodecyl sulfate polyacrylamide gel electrophoresis (SDS-PAGE) gels. Separated proteins were transferred to polyvinylidene difluoride membranes, and the blots were probed with the primary antibodies and HRP-conjugated secondary antibodies using the chromogenic Western Blot Immunodetection Kit (WesternBreeze Invitrogen Corp., Carlsbad, CA) according to the manufacturer's instructions. The primary mouse anti-TM antibody TM311 (Sigma-Aldrich), which detects sarcomeric tropomyosin isoforms, and the monoclonal anti-GFP antibody (JL-8) (Clontech), which recognizes GFP were used. Bands intensity was quantified through densitometric analysis. The expression level of α-tubulin was used as loading control. After WB analysis of tropomyosin isoforms and GFP, the membrane was stripped and incubated overnight at 4°C with a rabbit anti-alpha-tubulin antibody (ab4074) (Abcam), washed 3×5 min in wash buffer, and incubated thereafter for 1 h with horseradish peroxidase-labeled goat anti-mouse antibody (1∶2,500; Pierce, Rockford, IL). The immunoreaction was detected with SuperSignal West Femto chemiluminescent substrate (Pierce), and captured with a Fujifilm LAS 4000 CCD camera, and analysed with Multi Gauge v3.1 software.

### P62 immunofluorescence analysis of transfected cells

Human and mouse C2C12 myoblasts transfected with the WT-β-TM_EGFP_, E41K-β-TM_EGFP_ and E122K-β-TM_EGFP_ constructs were fixed and permeabilised as describe above. Cells were incubated with primary mouse monoclonal anti-p62/SQSTM1 (D-3), diluted 1∶1000 (Santa Cruz Biotechnology, Santa Cruz, CA) for 20 min at room temperature, followed by a far red DyLight® 649 Horse Anti-Mouse IgG (DI-2649) secondary antibody (Vector Laboratories, Inc, Burlingame, CA), diluted 1∶1000, and Alexa Fluor® 555 Phalloidin for 20 min. The slides were mounted using Prolong® Gold antifade reagent with DAPI to highlight cell nuclei and labeled cells were examined using an LSM 700 inverted Axio Observer.Z1 microscope, using a Plan-Apochromat 63×/1.4 Oil DIC. Images were processed using Photoshop software (Adobe, USA).

### Multiple sequence alignment

To assess the evolutionary conservation of beta tropomyosin in humans and mice, the entire amino acids were aligned using ClustalW (http://www.ebi.ac.uk/Tools/msa/clustalw2/).

## Results

**Figure 1:**
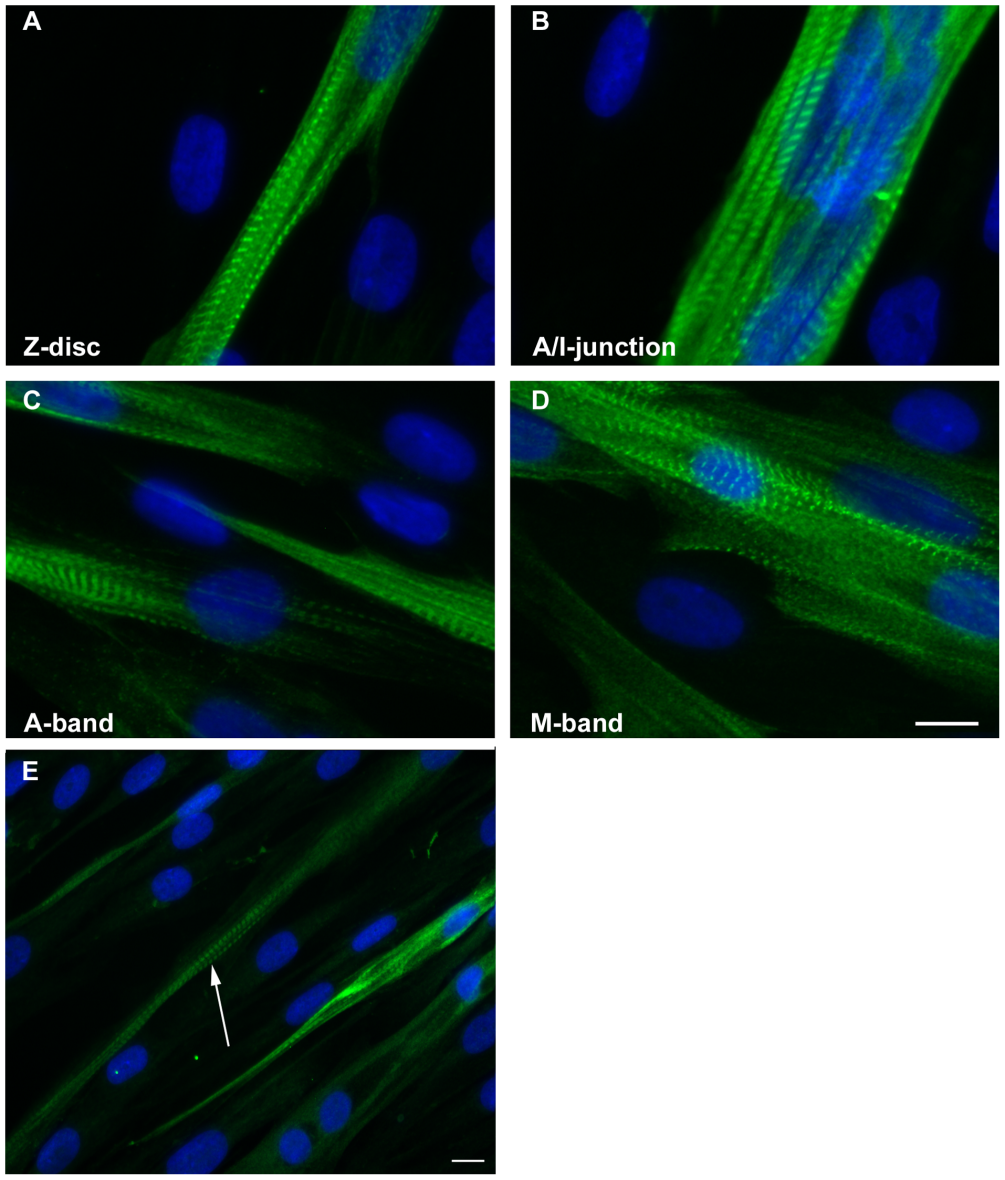
Immunofluorescence micrographs of labeled three-day human myotube culture developing the cross-striated myofibrils. Monoclonal anti-titin antibodies recognising titin epitopes in the Z-disk (A), A/I junction (B), A-band (C) and M-band (D). Three-day differentiated human culture cells were identified in an advanced developed state by a striated staining pattern for slow and fast IIa MyHCs, located in the A-band (E; arrow). Nuclei are labeled with DAPI (blue). Cells were imaged using a Zeiss Axio Observer microscope. Scale bar  = 10 µm.

We aimed to investigate the effects of five different β-TM mutations on myofibril organisation and to examine the localisation of mutant β-TMs within the cytoskeleton and myofilament. To accomplish this, we generated a C-terminal EGFP-tagged β-TM fusion gene, which was expressed in human mononuclear myoblasts and mouse C2C12. In addition, transfected myoblasts were then differentiated into multinuclear myotubes for three to six days. The ability to differentiate human myoblasts into myotubes and the development of cross-striated myofibrils were examined with a panel of titin antibodies. Myotubes in an advanced developed state were identified by elongated shape and multiple nuclei. The mature sarcomeric cross-striated pattern of the differentiated cells and their myofibrils was confirmed by immunostaining with titin. As in mature muscle cells, the four well-defined sarcomeric structures including Z-disk, A/I junction, A-band and M-band were clearly distinguishable in our human muscle tissue culture (Fig. 1A–D). After three days of differentiation, the vast majority of human myotubes showed positive immunostaining with embryonic and fetal MyHCs. This indicated that three-day differentiated human culture cells predominantly express the developmental variants of MyHC, the embryonic and fetal isoforms. However about 30% of the myotubes showed positive immunoreactivity with antibody against fast IIa and slow-β/cardiac MyHCs (Fig. 1E). They were identified in an advanced developed state by elongated shape, multiple nuclei and a striated staining pattern for MyHC, located in the A-band (Fig. 1E).

To examine the expression of β-TM in human and C2C12 myoblasts and one- to five-day differentiated myotubes, immunofluorescence analysis was performed. In contrast to human myoblasts, the expression of β-TM was lacking in proliferating myoblasts of C2C12 and its expression was only detected in the differentiated multinucleated cells (data not shown).

At least ten duplicated transfection experiments were performed per construct, with similar results. The transfection efficiency, morphology and cytoplasmic distribution of the fluorescently tagged β-TMs were examined in light and confocal microscopes. In addition, fluorescent phalloidin was used to probe for filamentous actin to examine the integration and localisation of mutant β-TMs into endogenous filament actin. The scoring of transfected cells indicated a high-efficiency transfection, which ranged between 60% and 70% of human myoblasts and 70% and 80% of C2C12 myoblasts. The differentiation efficiency was not affected by the transfection of WT or mutant β-TMs. However, the differentiation of transfected cells with mutant TM affected human cell viability.

The behaviour of myopathy-related β-TM mutants in human myoblasts resulted in a wide range of various phenotypes, which generally changed upon differentiation to myotubes (Table S1). The accumulation of mutant β-TM_EGFP_ in the perinuclear and peripheral areas of the myoblasts and differentiated cells was the striking feature in C2C12 (Table S1). The transfected myoblasts and myotubes were classified into categories depending on β-TM incorporation and the induced phenotypes (Table S2 and Diagram S1).

### Human cells transfected with mutant β-TM_EGFP_ appeared cytopathic, with aggregates and an unorganised filamentous structure of endogenous actin

**Figure 2:**
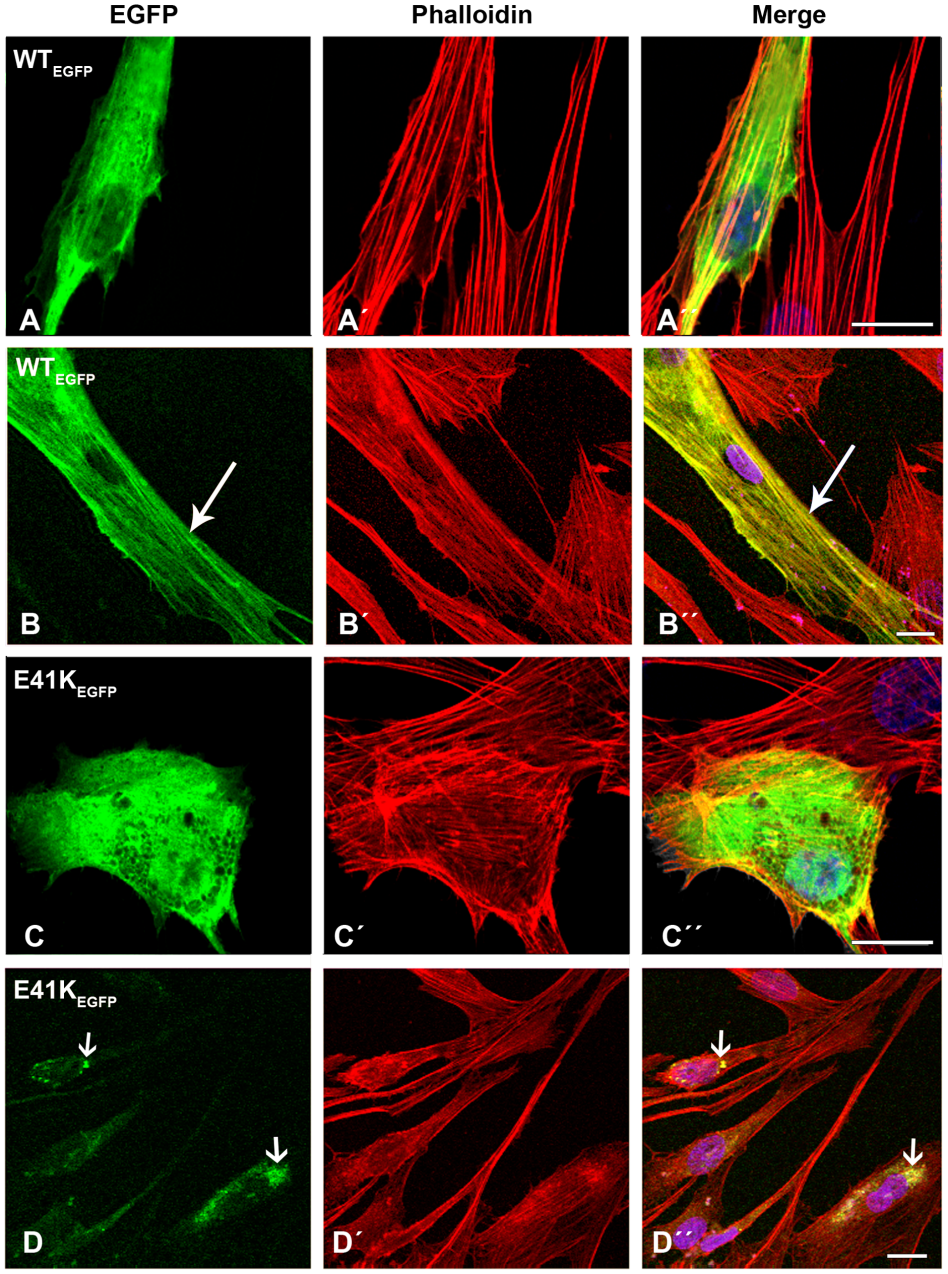
Expression of wild-type β-TM_EGFP_ and E41K-β-TM_EGFP_ in human cells. (A–B) Wild-type β-TM_EGFP_ and (C–D) E41K-β-TM_EGFP_ mutants were transfected in human (A and C) myoblasts and (B and D) myotubes differentiated for three to six days and labeled with TRITC-phalloidin (red) and DAPI (blue) to highlight cell nuclei. (A) WT-β-TM was expressed in human myoblasts or (B) myotubes and incorporated well into endogenous filamentous actin as visualised with phalloidin (long arrows). The E41K-β-TM mutant induced diffuse intranuclear and cytoplasmic clouds in transfected myoblasts (C–Ć ´’’) and perinuclear aggregates in myotubes (D–D´ ´’’; short arrows). Confocal microscopy was performed using a Zeiss LSM 510 Meta confocal microscope or an LSM 700 inverted Axio Observer.Z1 microscope. Scale bar  = 10 µm.

The transfection of wild-type β-TM resulted in the cytoplasmic localisation of the fusion protein and efficient incorporation into stress fibres and filamentous structures in the vast majority of human myoblasts (Fig. 2A–A”). To characterise the behaviour of β-TMs in a differentiated myotube system, transfected myoblasts were differentiated for three to six days. The ability of WT and mutant β-TMs to contribute to the sarcomeric thin filament was examined. WT-β-TM_EGFP_ predominantly localised in sarcomeric thin filaments of human elongated and multinucleated differentiated myotubes and the filamentous structures appeared well defined and organised (Fig. 2B–B”; long arrows).

**Figure 3:**
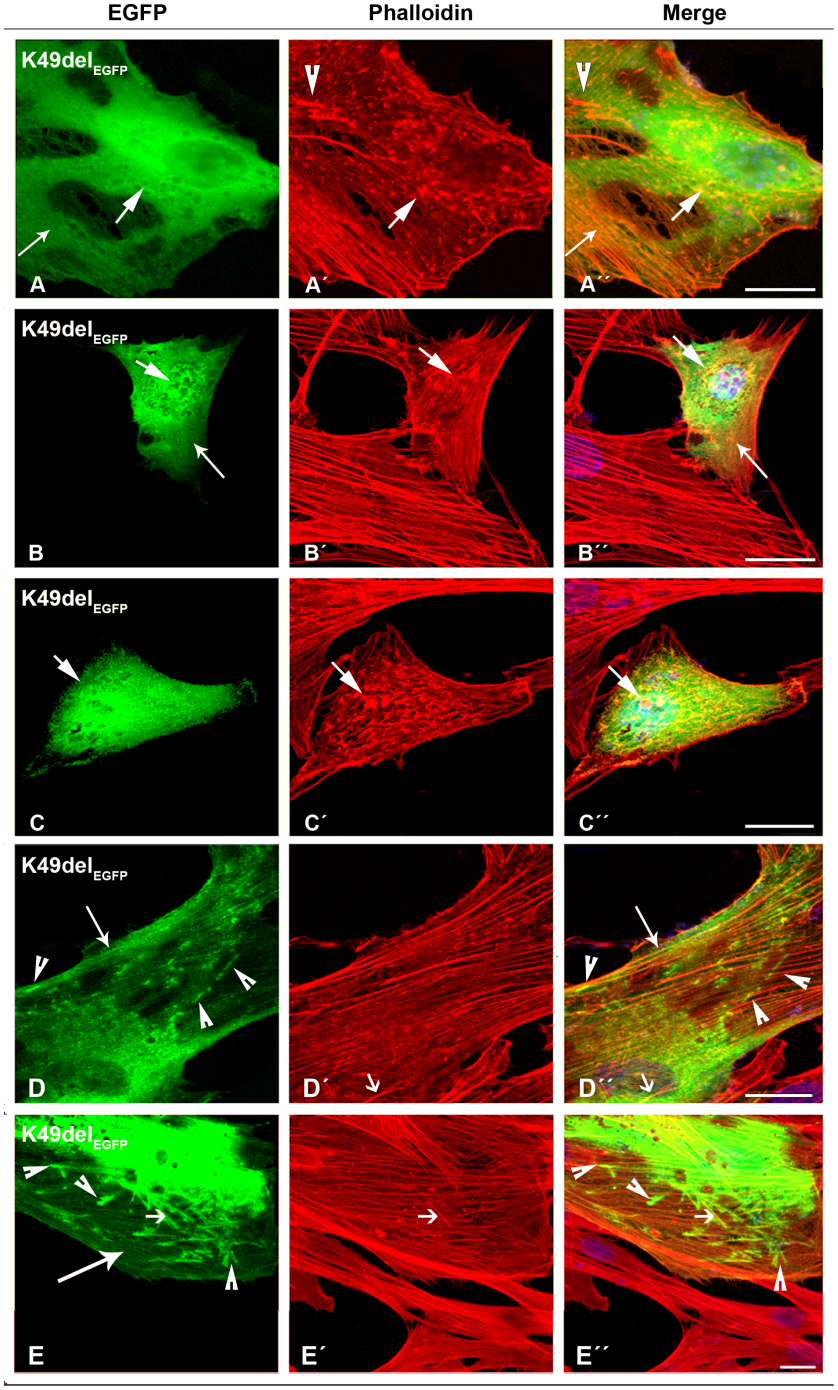
The expression of K49del-β-TM_EGFP_ in human cells. (A–D) The K49del-β-TM_EGFP_ mutant was transfected in human myoblasts and (E) myotubes and labeled with TRITC-phalloidin (red) and DAPI (blue) to highlight cell nuclei. The K49del-β-TM co-localised with cytoplasmic and nuclear aggregates of endogenous actin, detectable with phalloidin labeling (A–C”; short arrows). It produced clouds around the nucleus (A and D) but was also incorporated into stress fibres and filamentous lamellipodia (A and A”, B and B”, and D and D”; long arrows). In addition, it induced thickened filamentous structures of endogenous actin (A’–A”; arrow heads). The K49del-β-TM produced small, long rod-shaped intranuclear structures. A subset of aggregates labeled with phalloidin but was not detectably composed of EGFP-tagged mutant K49del-β-TM (D’–D”; short arrows). Frequently, cytoplasmic thickened filamentous structures with no co-localisation of F-actin were found in myoblasts transfected with K49del-β-TM (D and D”; arrow heads). The K49del-β-TM_EGFP_ mutant produced rod-shaped filamentous actin, cytoplasmic aggregates and cloud patterns in human myotubes (E). The rod-shaped structures were labeled with phalloidin, indicating co-localisation with F-actin (E–E”; short arrows). Cytoplasmic thickened filamentous structures were also observed (E and E”; arrow heads). The K49del-β-TM_EGFP_ mutant was also incorporated into filamentous actin (E; long arrow). Confocal microscopy was performed using a Zeiss LSM 510 Meta confocal microscope or an LSM 700 inverted Axio Observer.Z1 microscope. Scale bar  = 10 µm.

**Figure 4:**
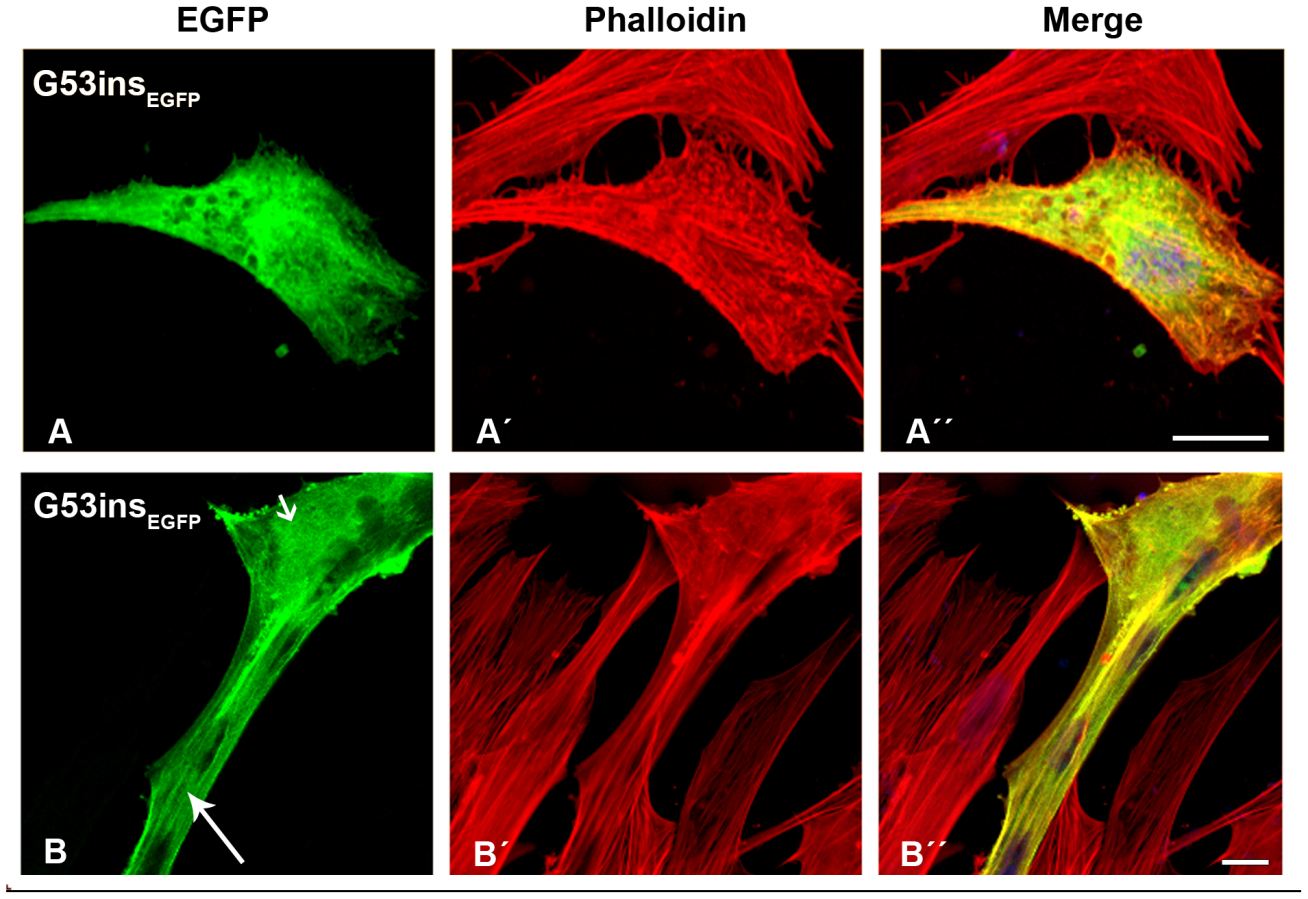
The expression of G53ins-β-TM_EGFP_ in human cells. The G53ins-β-TM_EGFP_ mutant was transfected in human (A) myoblasts and (B) myotubes and labeled with TRITC-phalloidin (red) and DAPI (blue) to highlight cell nuclei. The G53ins-β-TM_EGFP_ mutant produced delocalisation and endogenous actin aggregates in human myoblasts, labeled with phalloidin (A–A”). The cells transfected with the G53ins-β-TM mutant differentiated into myotubes in an advanced, developed state, identified by their elongated shape and multiple nuclei (B–B”). Transfected myotubes showed good integration of the mutant TM into sarcomeric structures (B; long arrow). The G53ins mutant produced diffuse cytoplasmic labeling at the far end of the myotubes (B; short arrow)_._ Confocal microscopy was performed using a Zeiss LSM 510 Meta confocal microscope or an LSM 700 inverted Axio Observer.Z1 microscope. Scale bar  = 10 µm.

In contrast, cells transfected with mutant β-TM_EGFP_ appeared cytopathic (Tables S1 and S2). The E41K-β-TM_EGFP_ mutant induced significant levels of diffuse localisation in transfected myoblasts and its incorporation within stress fibres in the cytoplasm was reduced (Fig. 2C–C”). The behaviour of the E41K-β-TM_EGFP_ mutant changed upon differentiation and it frequently formed perinuclear aggregates in myotubes, which were co-localised with F-actin, indicated by phalloidin labeling (Fig. 2D and D”; short arrows). In human myoblasts, K49del-β-TM_EGFP_ was incorporated in clouds at random locations in the nucleus and cytoplasm (Fig. 3A–D). It was co-localised in phalloidin-labeled cytoplasmic and nuclear aggregates (Fig. 3A–C”; short arrows). The K49del-β-TM_EGFP_ also produced thickened filaments (Fig. 3A’–A”; arrow heads) and intranuclear rod-shaped structures only detectable with phalloidin labeling (Fig. 3D’–D”; short arrows). Frequently, cytoplasmic thickened filamentous structures with no co-localisation of F-actin were found in myoblasts transfected with K49del-β-TM_EGFP_ (Fig. 3D and D”; arrow heads). The lack of co-labeling in these filaments could indicate either accumulations that were composed of unpolymerised actin or that they were not accessible to phalloidin. In addition, the K49del-β-TM_EGFP_ incorporated into stress fibres and filamentous lamellipodia (Fig. 3A and A”, 3B and B”, and 3D and D”; long arrows). Similar findings were observed in K49del-β-TM_EGFP_-transfected myotubes. The rod-shaped filaments, cytoplasmic aggregates and cloud-patterned structures were the common features in the myotubes. The rod-like structures were labeled with phalloidin, indicating co-localisation with F-actin (Fig. 3E–E”; short arrows). Notably, the intranuclear rod-shaped structures disappeared upon differentiation and they were instead found in cytoplasm. In differentiated cells, the cytoplasmic rod-shaped structures appeared to be the dominant phenotype. Like myoblasts, in many of the myotubes, cytoplasmic thickened filamentous structures with no co-localisation of F-actin were observed (Fig. 3E and E”; arrow heads). The K49del-β-TM_EGFP_ incorporated well into endogenous actin filaments in peripheral areas of the transfected myotubes, as observed in the myoblasts (Fig. 3E; long arrow). The most striking abnormality of G53ins-β-TM_EGFP_ mutant in human myoblasts was the formation of endogenous actin aggregates and the poor incorporation into the filamentous structure of stress fibres (Fig. 4A–A”). The localisation of G53ins-β-TM_EGFP_ changed considerably upon differentiation and transfected cells differentiated into myotubes in a developed state, identified by the elongated shape and multiple nuclei (Fig. 4B–B”). Transfected myotubes showed good integration of the mutant G53ins-β-TM_EGFP_ into sarcomeric structures (Fig. 4B; long arrow). However, the G53ins-β-TM_EGFP_ mutant produced diffuse cytoplasmic labeling at the far end of the myotubes (Fig. 4B; short arrow)_._

**Figure 5:**
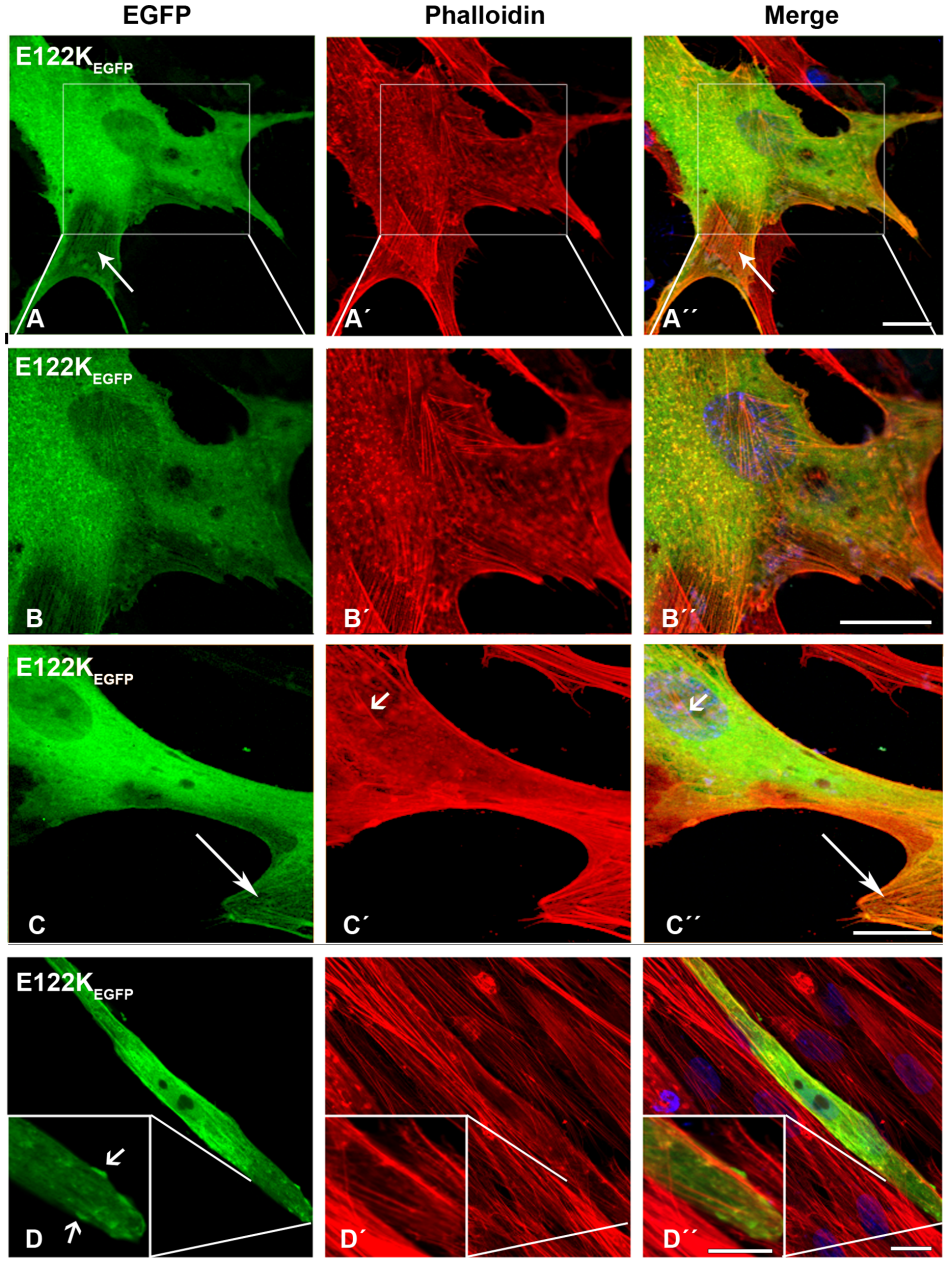
The expression of E122K-β-TM_EGFP_ in human cells. The E122K-β-TM_EGFP_ mutant was transfected in human (A–C) myoblasts and (D) myotubes and labeled with TRITC-phalloidin (red) and DAPI (blue) to highlight cell nuclei. The E122K-β-TM_EGFP_ mutant formed aggregates of endogenous actin in human myoblasts (A–A”; inset (B–B”)). Moreover, the E122K-β-TM_EGFP_ incorporated in clouds with an unorganised filamentous structure (C–C”). E122K-β-TM_EGFP_ formed intranuclear rods in human myoblasts that were only detectable with phalloidin labeling (C–C”; short arrows). It also integrated well with stress fibres (A–A” and C–C”; long arrows). The transfection of the E122K-β-TM_EGFP_ construct generally resulted in the less well-defined phalloidin labeling of actin filaments at the far end of the transfected myotubes with the appearance of small rod-like structures located at the periphery (D–D”; inset, short arrows). The rod-shaped structures did not label with phalloidin, indicating that they were not accessible to phalloidin or were not composed of filamentous actin. Confocal microscopy was performed using a Zeiss LSM 510 Meta confocal microscope or an LSM 700 inverted Axio Observer.Z1 microscope. Scale bar  = 10 µm.

The E122K-β-TM_EGFP_ mutant behaved in roughly the same way as the K49del-β-TM_EGFP_ mutant (Fig. 5A–C”). E122K-β-TM_EGFP_ formed intranuclear rods in human myoblasts that were only detectable with phalloidin labeling (Fig. 5C’–C”; short arrows). The transfection of the E122K-β-TM_EGFP_ construct resulted in the less well-defined phalloidin labeling of actin filaments at the far end of the transfected myotubes (Fig. 5D–D”). As in the myoblasts, the E122K-β-TM_EGFP_ mutant induced rod-like structures in the myotubes, although they appeared smaller and were located at the periphery of the far end of the cells (Fig. 5D–D”; inset). These structures were not detectably labeled with phalloidin.

**Figure 6:**
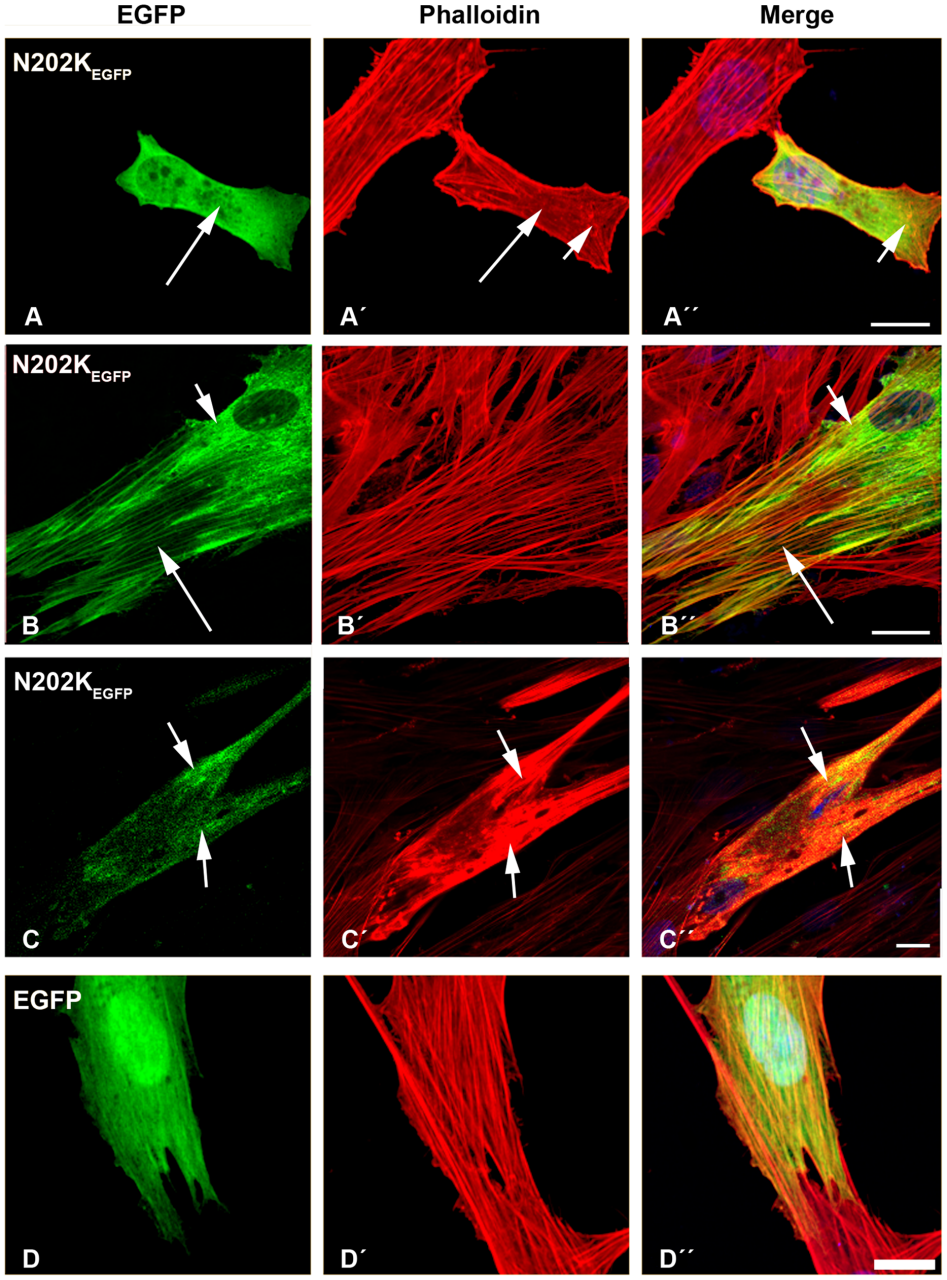
The expression of E202K-β-TM_EGFP_ and empty EGFP constructs in human cells. The E202K-β-TM_EGFP_ mutant was transfected in human (A–B) myoblasts and (C) myotubes. (D) Empty EGFP construct was transfected in myoblasts. Cells were labeled with TRITC-phalloidin (red) and DAPI (blue) to highlight cell nuclei. The transfection of human myoblasts with mutant N202K-β-TM induced the diffuse cytoplasmic labeling of stress fibres (A–A’; long arrows) and small phalloidin-labeled aggregates in the cytoplasm (A’ and A”; short arrows). Mutant N202K-β-TM formed clouds around the nucleus (B and B”; short arrows), in addition to a well-defined organised filamentous structure of stress fibres (B–B”; long arrows). In many N202K-β-TM_EGFP_-transfected myotubes, more marked changes in actin structures were observed (C–C”). A large accumulation of mutant N202K-β-TM with the co-localisation of polymerised actin appeared, suggesting the disruption of endogenous actin filaments (C–C”; short arrows). Human myoblasts transfected with empty EGFP vector formed well-organised filamentous structures (D–D”). Confocal microscopy was performed using a Zeiss LSM 510 Meta confocal microscope or an LSM 700 inverted Axio Observer.Z1 microscope. Scale bar  = 10 µm.

The transfection of human myoblasts with the N202K-β-TM_EGFP_ mutant induced the diffused cytoplasmic labeling of stress fibres (Fig. 6A–A’; long arrows) with small aggregates in the cell cytoplasm that labeled with phalloidin (Fig. 6A ´ and A”; short arrows). A subset of mutant N202K-β-TM_EGFP_ formed clouds around the nucleus and in the cytoplasm (Fig. 6B and B”; short arrows). After differentiation, a more severe phenotype was observed. In many myotubes, noticeable changes in actin structures could be found. Sarcomeric thin filaments appeared to be extremely thickened, resulting in a huge accumulation of polymerised actin, suggesting the disruption of endogenous actin filaments with the co-localisation of the mutant N202K-β-TM_EGFP_ (Fig. 6C–C”; short arrows).

To establish that the EGFP tagging of TM did not cause the formation of TM accumulations and abnormal phenotypes of transfected cells, transfection with empty EGFP vector was performed. An analysis of human myoblasts transfected with empty EGFP vector clearly demonstrated the formation of the organised filamentous structures (Fig. 6D–D”). Findings such as aggregation or accumulation were not observed in the cells transfected with empty EGFP vector. Similar results were observed in myoblasts transfected with empty EGFP and then differentiated into myotubes. In addition, we performed a control study using the lipid exclusively to determine that abnormal structures and the reduced labeling of phalloidin were not toxic functions of the lipofectamine. An analysis of these cells demonstrated the normal formation of filamentous structures without the appearance of aggregates or accumulations.

**Figure 7:**
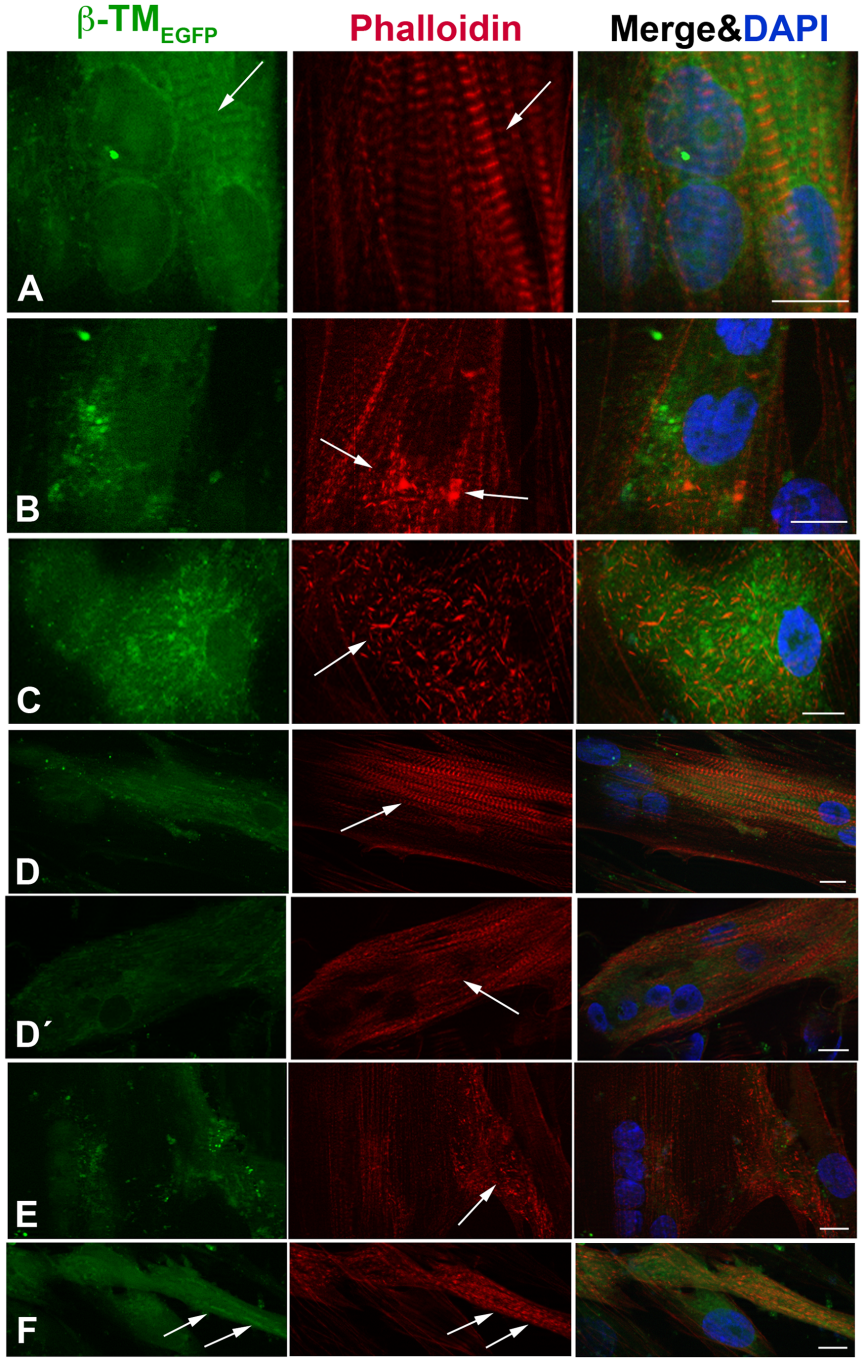
The expression of WT and mutant β-TMs-_EGFP_ in human myotubes. WT and mutant β-TMs were transfected in human myotubes differentiated for three to six days and labeled with TRITC-phalloidin (red) and DAPI (blue) to highlight cell nuclei. (A) WT-β-TM was expressed in human myotubes and incorporated well into endogenous sarcomeric thin filaments as visualised with phalloidin (arrows). (B) The E41K-β-TM mutant induced diffuse perinuclear aggregates in myotubes (arrows). (C) The K49del-β-TM_EGFP_ mutant produced cytoplasmic rod-shaped filamentous actin in human myotubes. (D) The cells transfected with G53ins-β-TM mutant differentiated into myotubes, showed the integration of the mutant TM into sarcomeric structures and developed the cross-striated myofibrils in an advanced and developed state (arrow). (D’) The G53ins mutant produced diffuse cytoplasmic labeling at the far end of the transfected myotubes (arrow)_._ (E) The transfection of the E122K-β-TM_EGFP_ construct generally resulted in the appearance of cytoplasmic rod-like structures located at the periphery of the transfected myotubes (arrow). (F) The cells transfected with N202K-β-TM_EGFP_ differentiated into myotubes, showed an accumulation of mutant N202K-β-TM, co-localisation with polymerised actin (arrows). Cells were imaged using a Zeiss Axio Observer microscope. Scale bar  = 10 µm.

The morphology, integration and localisation of WT and mutant β-TMs into endogenous sarcomeric actin filament were examined in transfected and differentiated human myotubes by inverted light microscope (Fig. 7). Six-day transfected and differentiated human culture cells were identified in an advanced developed state by multiple nuclei and a striated staining pattern for actin. The EGFP-tagged WT-β-TM was located in the sarcomeric thin filaments (Fig. 7A; arrows).

**Figure 8:**
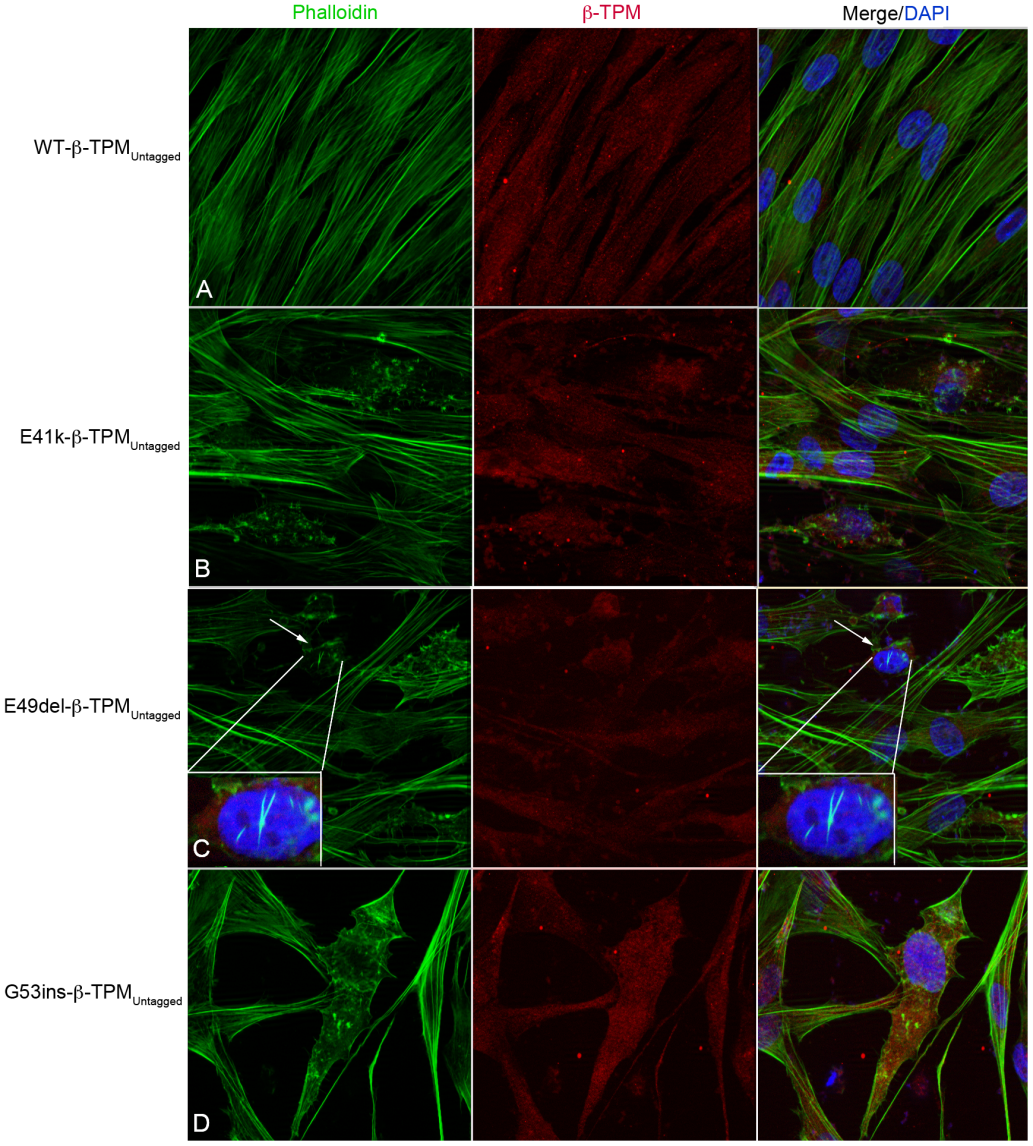
Untagged β-TM constructs form abnormal aggregates in human myoblasts. Human myoblasts transfected with untagged WT-, E41K-, K49del- and G53ins-β-TM constructs and labeled with phalloidin (green) and β-TM (red) and DAPI (blue) to highlight cell nuclei. Stress fibres appeared well aligned in human myoblasts transfected with WT-β-TM (A). Abnormal aggregates were observed in human myoblasts transfected with untagged E41K-, K49del- and G53ins-β-TM constructs (B–D). Intranuclear rod-shaped aggregates labeled by phalloidin are detected in human myoblasts transfected with the untagged K49del-β-TM construct, demonstrating that intranuclear aggregation is an inherent property of K49del mutation and does not result from EGFP-tagging (C; arrows).

To provide further evidence that the formation of aberrant structures and the reduced labeling of phalloidin to the filamentous structures were not the result of EGFP fusion, human myoblasts were transfected with untagged WT-β-TM and three selected mutant constructs (E41K-, K49del- and G53ins-β-TM). Human myoblasts transfected with the three untagged mutant β-TM constructs resulted in cytopathic features, similar to the corresponding EGFP-tagged constructs. Unlike mutants, no evidence of aggregates or rod-shaped structures was observed using an untagged WT-β-TM construct (Fig. 8).

### β-TM_EGFP_ mutants differently incorporate into cytoskeleton and sarcomeric filaments

We examined the relative expression levels of exogenous β-TM and the ability of β-TM mutants to incorporate into the filamentous cytoskeleton of transfected human myoblasts and six-day differentiated myotubes. Triplicate wells were harvested 16–18 h after replating (myoblasts) and 6 days of differentiation (D6).

To examine expression levels of WT and mutant exogenous β-TM, we performed WB analysis in transfected cells using an anti-GFP antibody. We then examined the relative expression levels of exogenous β-TM and endogenous TM isoforms in transfected human myoblasts and differentiated myotubes (D6) using TM311 antibody, which recognizes the sarcomeric tropomyosin isoforms. Bands of equal intensity were observed for exogenous WT and various β-TM mutants in transfected cells (data not shown). This indicates equal levels of expression from the various β-TM constructs in repeated transfection experiments.

**Figure 9:**
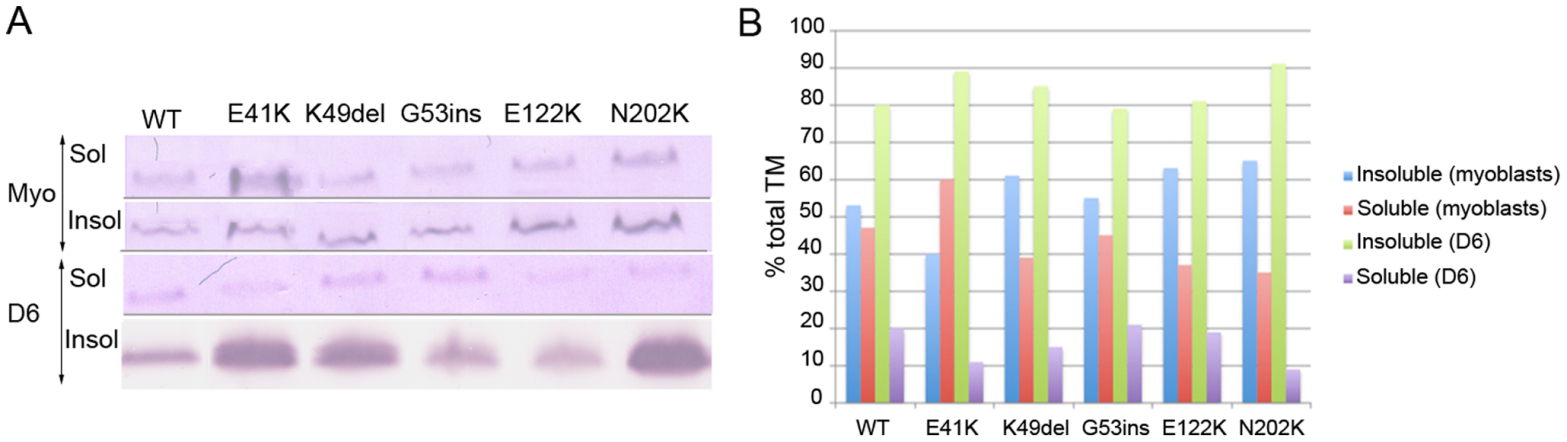
Incorporation of β-TM mutants into cytoskeleton and sarcomeric filaments in cultured human myoblasts and differentiated myotubes. (A) Western blot images from a single experiment showing levels of β-TM_EGFP_ within the insoluble (Insol) and soluble (Sol) protein pools in myoblasts (Myo) and in six-day differentiated cultures (D6). Bands intensity was quantified through densitometric analysis. The mean data from triplicate experiments is shown in (B).

In addition, the relative levels of mutant β-TM_EGFP_ present in the soluble (representing unbound protein and short filaments) and insoluble (representing large cytoskeletal and sarcomeric filaments and also protein aggregates) protein pools in undifferentiated myoblasts and differentiated myotubes (D6) were examined (Fig. 9). While in myoblasts, WT-β-TM_EGFP_ was present at roughly equal levels in insoluble and soluble fractions, there was a increase in levels of insoluble β-TM (80%) in differentiated myotubes. E41K-β-TM_EGFP_ contributed less efficiently to insoluble filaments, compared with WT-β-TM_EGFP_, localizing mainly to the soluble protein fraction (60% of total TM) in myoblasts. However, there was an increase to 89% in the proportion of E41K-β-TM in the insoluble protein pool in six-day differentiated myotubes. This is consistent with our morphological findings for E41K-β-TM_EGFP,_ which showed its reduced incorporation within stress fibres in the cytoplasm and formation of perinuclear aggregates co-localised with F-actin in myotubes. K49del-β-TM_EGFP_, G53ins-β-TM_EGFP_, E122K-β-TM_EGFP_ and N202K-β-TM_EGFP_ constructs behaved similarly, with approximately 55–65% of the expressed β-TM detected within the insoluble cytoskeleton in myoblasts, and increasing to 79–91% in six-day differentiated myotubes.

### Intense EGFP aggregates co-localised with F-actin in the peripheral area of the C2C12 cells transfected with mutant β-TM_EGFP_

**Figure 10:**
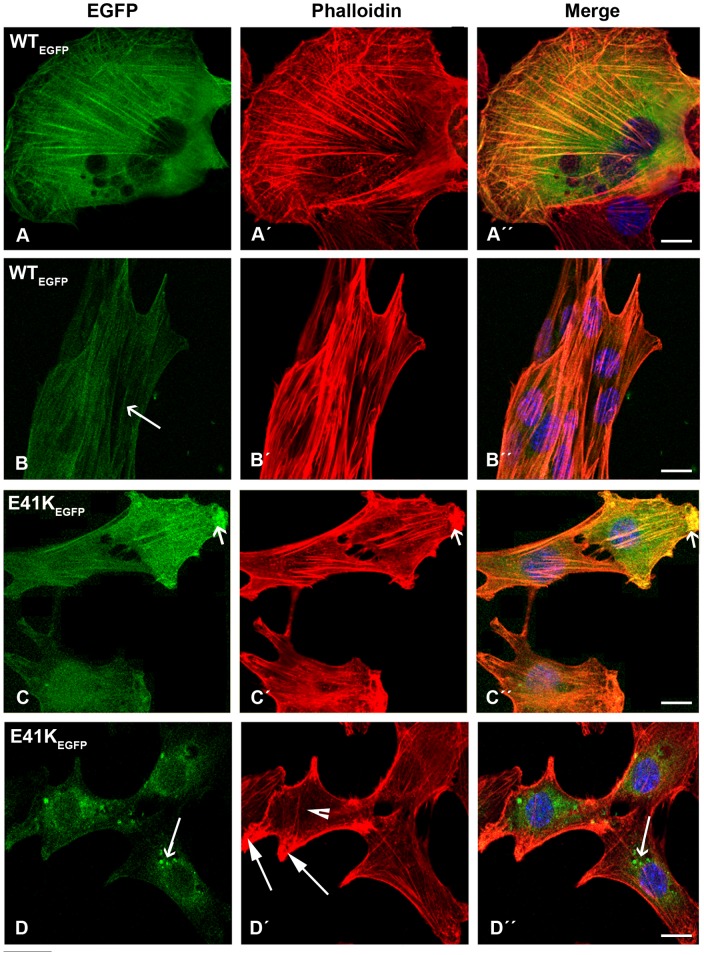
The expression of wild-type-β-TM_EGFP_ and E41K-β-TM_EGFP_ in C2C12. (A–B) Wild-type β-TM_EGFP_ and (C–D) E41K-β-TM_EGFP_ mutant were transfected in (A and C) the myoblasts and (B and D) myotubes differentiated for three to six days and labeled with TRITC-phalloidin (red) and DAPI (blue) to highlight cell nuclei. WT-β-TM expressed in C2C12 (A) myoblasts or (B) myotubes incorporated well into C2C12 stress fibres (A) and endogenous filamentous actin (B; long arrow) as visualised with phalloidin. The E41K-β-TM mutant appeared to be diffused in transfected myoblasts and intense EGFP aggregates co-localised with endogenous actin in the peripheral area of the cells (C–C”; short open arrows). The E41K-β-TM mutant formed perinuclear aggregates in myotubes that did not show phalloidin labeling (D–D’’; long open arrows). C2C12 cells appeared as fused myoblasts rather than differentiated myotubes in six-day differentiated cultures. Confocal microscopy was performed using a Zeiss LSM 510 Meta confocal microscope or an LSM 700 inverted Axio Observer.Z1 microscope. Scale bar  = 10 µm.

**Figure 11:**
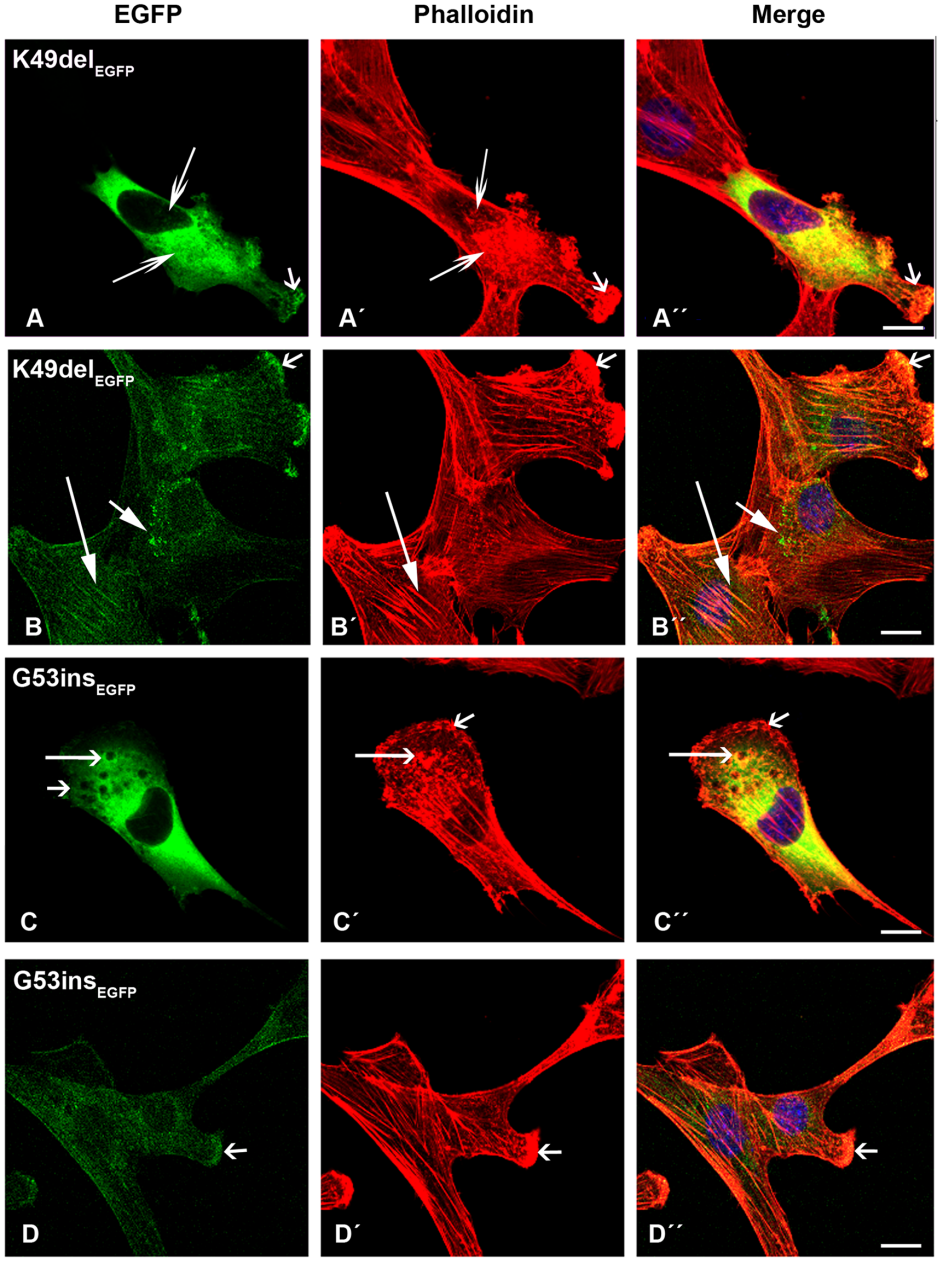
The expression of K49del and G53ins β-TM-EGFP in C2C12. The K49del β-TM-EGFP mutant was transfected in (A) myoblasts and (B) myotubes and labeled with TRITC-phalloidin (red) and DAPI (blue) to highlight cell nuclei. (A) The K49del-β-TM mutant was mislocalised and showed the diffuse labeling of phalloidin in transfected myoblasts. It induced nuclear and cytoplasmic aggregates (A–A’; long open arrows). The K49del-β-TM mutant produced intense EGFP aggregates, co-localised with endogenous actin in the peripheral area of both myoblasts (A–A”; short arrows) and differentiated cells (B–B”; short open arrows), in addition to perinuclear aggregates in differentiated C2C12 (B and B”; short closed arrows). (B) The K49del-β-TM mutant showed some integration with actin filaments of differentiated C2C12 (B–B”; long arrows). The G53ins mutant appeared mislocalised, showed the diffuse labeling of phalloidin in transfected myoblasts (C–C”) and formed cytoplasmic aggregates (C–C”; long open arrows). Intense aggregates in the peripheral area of both myoblasts (C–C”; short arrows) and differentiated cells (D–D”; short arrows) were observed. Confocal microscopy was performed using a Zeiss LSM 510 Meta confocal microscope or an LSM 700 inverted Axio Observer.Z1 microscope. Scale bar  = 10 µm.

**Figure 12:**
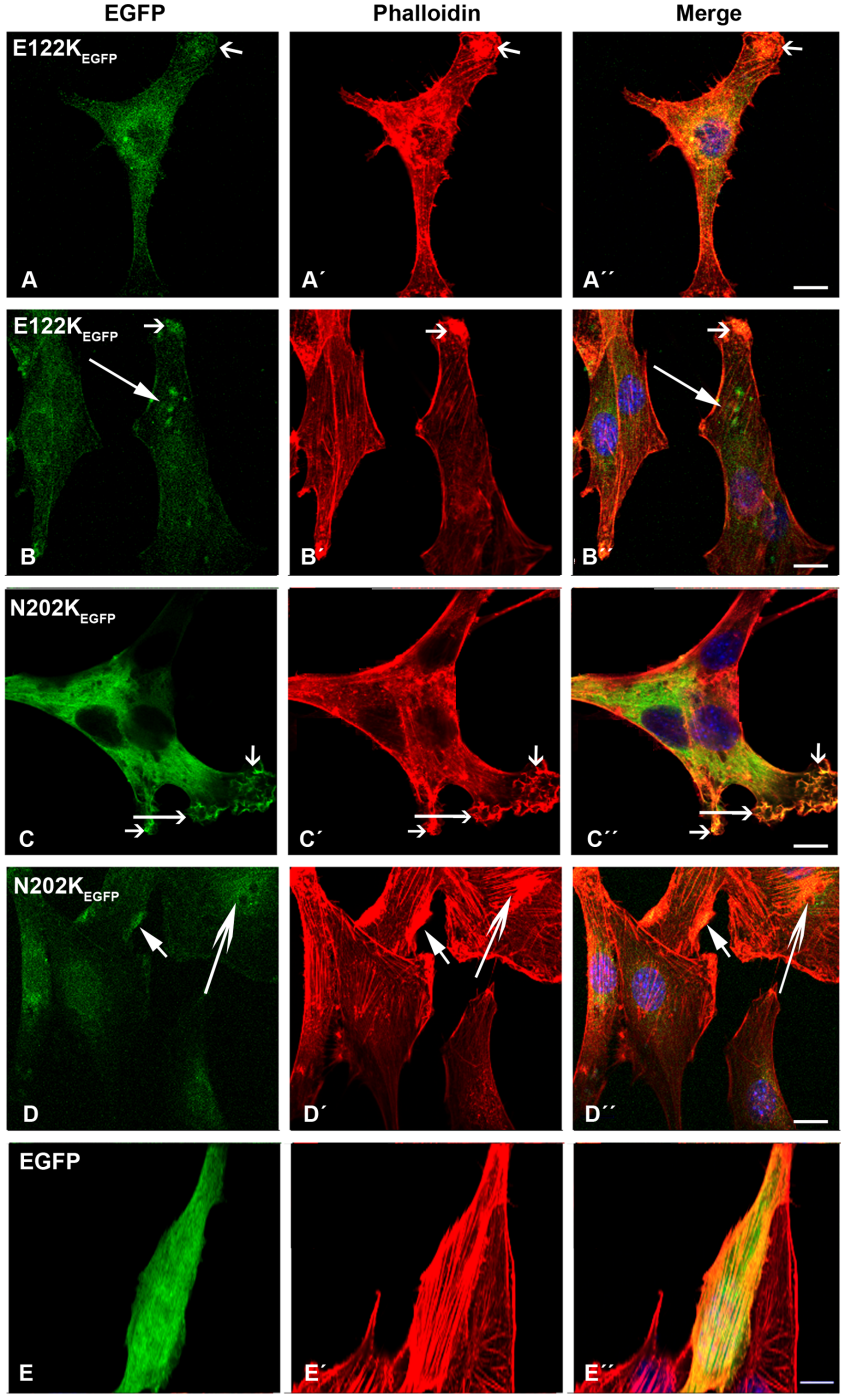
The expression of E122K and N202K-β-TM_EGFP_ and empty EGFP in C2C12. The cells were labeled with TRITC-phalloidin (red) and DAPI (blue) to highlight cell nuclei. The E122K-β-TM_EGFP_ mutant was transfected in (A) myoblasts and (B) myotubes. (A) The E122K-β-TM mutant was mislocalised and produced intense EGFP aggregates, co-localised with endogenous actin in the peripheral area of both myoblasts (A–A”; short arrows) and differentiated cells (B–B”; short arrows). The E122K mutant induced the perinuclear aggregates in differentiated C2C12 (B and B”; long arrows). C2C12 myoblasts transfected with N202K-β-TM_EGFP_ appeared cytopathic, with a thickened, ruffled cell surface (C–C”; long open arrows). Aggregates in the peripheral (D–D”; short closed arrows) and perinuclear (D–D”; long open arrows) areas of differentiated cells were detected. The transfection of C2C12 myoblasts with empty EGFP vector resulted in the formation of well-organised stress fibres (E–E”). Confocal microscopy was performed using a Zeiss LSM 510 Meta confocal microscope or an LSM 700 inverted Axio Observer.Z1 microscope. Scale bar  = 10 µm.

The ability of the WT and mutant β-TM_EGFP_ to contribute to stress fibres and thin filaments in C2C12 myoblasts and myotubes was examined. WT-β-TM_EGFP_ produced well-organised filamentous structures, which incorporated into C2C12 stress fibres (Fig. 10A–A”) and the sarcomeric thin filament of multinucleated differentiated cells (Fig. 10B; long open arrow). Instead, intense EGFP aggregates co-localised with endogenous actin, mainly in the peripheral area of the cells, were the most striking abnormality produced by all β-TM_EGFP_ mutants in C2C12 myoblasts and myotubes (Fig. 10–12; short open arrows). In contrast to cells transfected with WT-β-TM_EGFP_ (Fig. 10B–B”), the differentiation of transfected C2C12 cells with mutant β-TM_EGFP_ was affected. In general, myoblasts appeared to be fused rather than elongated multinucleated differentiated myotubes. Mutant β-TM_EGFP_ constructs generally showed less well-defined phalloidin labeling in the majority of transfected C2C12 differentiated cells.

The E41K-β-TM_EGFP_ mutant induced the diffuse cytoplasmic labeling of stress fibres (Fig. 10C–C”). Like human myotubes, the E41K-β-TM_EGFP_ mutant produced perinuclear aggregates in differentiated C2C12 (Fig. 10D; long open arrow). However, unlike human myotubes, these aggregates did not show integration into actin filaments (Fig. 10D”; long open arrow). A subset of differentiated cells showed reduced cytoplasmic phalloidin labeling (Fig. 10D’; arrow head), although the peripheral area of the cells was markedly labeled (Fig. 10D’; long closed arrows). Myoblasts transfected with the K49del-β-TM_EGFP_ mutant showed abnormal localisation of mutant TM and diffuse labeling with phalloidin (Fig. 11A–A”). In addition, the K49del mutant formed nuclear and cytoplasmic aggregates in the myoblasts (Fig. 11A–A’; long arrows). This mutant showed some incorporation into the actin filaments of differentiated cells (Fig. 11B–B”; long closed arrows). The K49del-β-TM_EGFP_ mutant also produced perinuclear aggregates in differentiated C2C12 (Fig. 11B and B”; short closed arrows). Like the K49del-β-TM_EGFP_ mutant, myoblasts transfected with the G53ins-β-TM_EGFP_ showed abnormal localisation of mutant TM and diffuse labeling with phalloidin (Fig. 11C–C”), in addition to the formation of cytoplasmic aggregates (Fig. 11C–C”; long open arrows). The E122K-β-TM_EGFP_ mutant produced aggregates in the peripheral area of both myoblasts and differentiated cells (Fig. 12A–B”; short open arrows), in addition to the perinuclear aggregates in differentiated C2C12 (Fig. 12B and B”; long arrows). In addition to diffuse cytoplasmic localisation, a thickened and ruffled cell surface was produced in many C2C12 myoblasts transfected with N202K-β-TM_EGFP_ (Fig. 12C–C”; long open arrows). Peripheral (Fig. 12D–D”; short closed arrows) and perinuclear (Fig. 12D–D”; long arrows) aggregates were detected in N202K-β-TM_EGFP_ three- to six-day myotube cultures. The transfection of C2C12 myoblasts with empty EGFP vector resulted in the formation of well-organised stress fibres (Fig. 12E).

### Accumulation of p62 protein in E41K-β-TM_EGFP_ and E122K-β-TM_EGFP_ transfected cells

**Figure 13:**
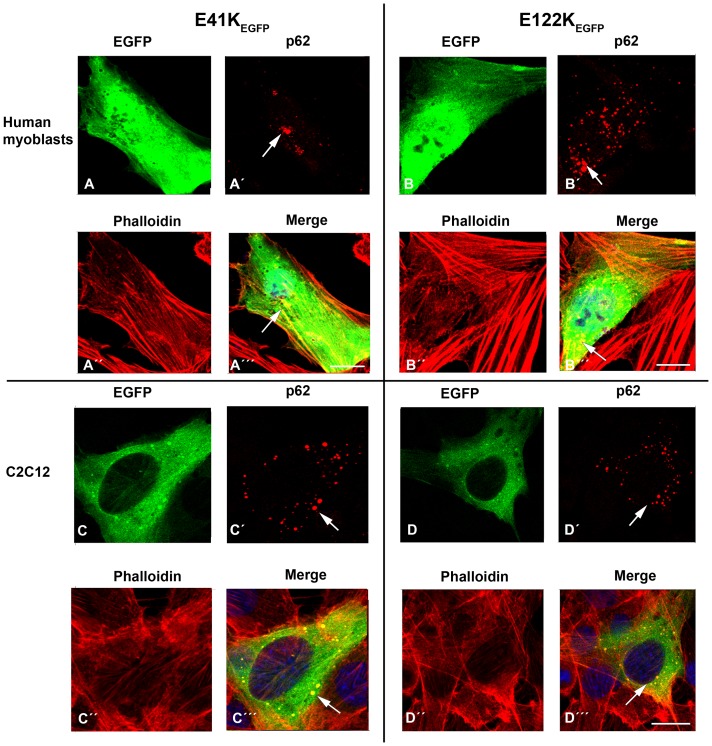
Immunofluorescent labeling of p62 in human myoblasts and C2C12 transfected with E41K-β-TM_EGFP_ and E122K-β-TM_EGFP_ constructs. The cells were labeled with TRITC-phalloidin (red) and DAPI (blue) to highlight cell nuclei. (A–A’”) The co-localisation of p62 (red, A’; arrow) in aggregates induced by the transfection of E41K-β-TM_EGFP_ in human myoblasts (A and A’”; arrows). (B–B’”) The co-localisation of p62 (red, B’; arrow) in aggregates induced by the transfection of E122K-β-TM_EGFP_ in human myoblasts (B and B’”; arrows). (C–C’”) C2C12 myoblasts transfected with E41K-β-TM_EGFP_ showed positive immunoreactivity with p62 (red, C’; arrow). (D–D’”) The co-localisation of p62 (red, D’; arrow) in aggregates induced by the transfection of E122K-β-TM_EGFP_ in C2C12 myoblasts (D and D’”; arrows). The p62 labeling closely resembled EGFP-positive protein aggregates in the transfected cells with mutant TM (i.e. yellow in the merged images A’”, B’”, C’” and D’”; arrows). Confocal microscopy was performed using an LSM 700 inverted Axio Observer.Z1 microscope. Scale bar  = 10 µm.

We examined the accumulation of p62, a polyubiquitinate-binding protein, in human and C2C12 myoblasts, transfected with WT- and mutant-β-TM_EGFP_ constructs. The E41K-β-TM_EGFP_ and E122K-β-TM_EGFP_ were selected, as virtually every cell transfected with these mutant constructs displayed aggregates or diffuse protein localisation. The accumulation of p62 was demonstrated using immunofluorescence staining and confocal microscopy. p62 was hardly detectable in human myoblasts and C2C12 cells overexpressing WT-β-TM_EGFP_. In contrast, labeling with antibody against p62 was apparent in both human myoblasts and C2C12 transfected with mutant TMs (Fig. 13). P62 was localised in the form of immunoreactive, small rounded aggregates, which closely resembled EGFP-positive protein aggregates in the transfected cells with mutant TM (Fig. 13; arrows).

## Discussion

The muscle tissues from patients with myopathy may often feature compensatory changes in other proteins in addition to the mutated one and this may complicate the interpretation of linking the morphological changes to a specific molecular defect. Transfected cultured cells can therefore be used as a cellular model to examine the sole consequences of amino acid alteration in the mutated protein and to find indications of the properties of the mutants that are likely to cause the morphological changes. The transfection of mouse C2C12 myoblasts and myotubes and NIH3T3 fibroblast cell lines has been used extensively to explore the molecular mechanisms underlying morphological abnormalities and muscle weakness associated with the gene mutations [36]–[41]. However, using human muscle tissue as an efficient host for gene transfer has so far been challenging. In order to mimic the physiological and cellular conditions of humans, we investigated the effects of *TPM2* mutations in human cultured cells. Using modified culturing and transfection methods, we were able to transfect human myoblasts relatively easily and found that exogenous TM expression was well tolerated. In addition, we differentiated transfected myoblasts into multinucleated myotubes to provide a more physiologically relevant context and to examine the incorporation of mutant β-TM isoforms into sarcomeric thin filaments, as the mutant TM resides within the cells for three to six days. Our culture technique resulted in the development of striated myotubes and the expression of the sarcomeric MyHC isoforms. Human three-day differentiated cultures were predominantly occupied by myotubes, showing a mature sarcomeric cross-striated pattern, with the four well-defined sarcomeric structures. In particular, the integration of M-band titin, which is a late stage in myofibrillogenesis [42], indicated the formation of a sarcomere structure *in vitro*. However, the differentiation of human transfected cells with mutant β-TM affected cell viability and many of the transfected myoblasts died prior to a sufficient differentiation level and the formation of myofibre-like cells with peripheral nuclei. In contrast, the survival rate for the WT-β?TM was as high as 70%. A different situation applied to C2C12. In general, the C2C12 appeared to be fused rather than differentiated in six-day myotube cultures, indicating that differentiation might be delayed. These results indicated that mutant β-TMs probably caused a dominant negative effect in the two cellular models by preventing either cell viability or differentiation ability. However, further studies are required to understand the impact of β-TM mutations on the differentiation efficiency of C2C12 and the viability of human myotubes.

In general, the appearance of the β-TM mutants differed pre and post differentiation. Most of the mutants showed various phenotypes ranging from mis- and diffuse protein localisation to the formation of thickened and large accumulations in transfected myoblasts. After several days of differentiation, they were either capable of contributing to the sarcomeric structure, as they showed good integration, or they were unable to incorporate into the actin thin filaments and formed huge aggregates. This may indicate that many of the effects observed in myoblasts are short-term *in vitro* defects rather than being of direct relevance to the molecular mechanisms of the mutant isoform.

The results from our human cellular model demonstrated that the E41K mutant was not readily integrated into stress fibres, as it produced diffuse localisation in the majority of undifferentiated myoblasts. On differentiation, the appearance of large perinuclear fluorescent filamentous actin aggregates and poor cytoplasmic integrations into the endogenous thin filaments occurred in human three- to six-day myotube cultures. These results are consistent with a reduced contribution of E41K-β-TM_EGFP_ to insoluble filaments, compared with WT-β-TM_EGFP_, in undifferentiated myoblasts and its predisposition for forming insoluble filaments in six-day differentiated myotubes. It is likely that the formation of the perinuclear aggregates was caused by an on-going process, similar to the time course of rod formation observed *in vivo* [8]. The E41K mutation is linked to the morphological features of cap disease and nemaline myopathy. The rods were not identified in muscle biopsies taken at different time points or within the family with the mutation [8]. The poor integration of the E41K mutant into stress fibres and sarcomeric actin filaments fits well with the predicted interaction defect. The E41K mutation is located at position *f* of the heptad repeat motif in the outer region of the helixes essential for the appropriate interaction with other proteins of sarcomeric thin filaments [8]. This is further indicated by the results of the *in vitro* study suggesting that E41K causes a reduced affinity for actin and abnormal actin-tropomyosin binding [23].

The fact that *TPM2* mutations cause morphologically different phenotypes suggests that the mutations differentially affect specific TM-binding interaction and function. It is also likely that the mutations invoke distinct structural changes within the α-helical coiled-coil dimer or thin filament. These predictions support our notion that the behaviour of the mutant β-TM in human cell cultures was mutation-specific and each mutant β-TM resulted in a similar cellular phenotype in repeated performances of the experiment. The K49del and E122K mutants, linked to cap disease and a non-specific congenital myopathy respectively, appeared mislocalised in most of the human myoblasts and formed large intranuclear rods that did not detectably contain the mutant β-TM isoforms. During differentiation, the K49del and E122K mutants instead formed cytoplasmic rod-shaped aggregates, composed of polymerised actin, suggesting that the differentiation of myoblasts caused the nuclear export of the rod aggregates. The appearance of the rod-like aggregates in both myoblasts and myotubes indicated either that the ability to form rods could be an inherent characteristic of these mutations or that it was a response by the cells to a certain pathological situation. In addition to rod formation, there were other features ranging from fragmented and shortened to long and thickened accumulations. It is possible to suggest that the capability of the K49del and E122K mutants to form rods and aggregates of different kinds in culture cells is linked to a specific molecular defect rather than any association with the nemaline bodies in patient muscles. The accumulation of K49del rather than integration into actin filaments might be the result of a reduced affinity for actin, as indicated by the results of an actin-binding assay [43]. In addition, the E122K mutant is situated at position *c* of the heptad repeat motif at the outer region of the helixes. A mutation in this position thus suggests the disruption of the interaction with other proteins such as actin [8].

The lack of aberrant accumulations in the main part of the differentiated cells transfected with the G53ins mutant may indicate that they were developed in an initial integration of mutant isoform into endogenous actin filaments, which eventually incorporate efficiently into thin filaments, or that they were short-term *in vitro* defects. However, the distinct localisation of the G53ins mutant protein in the myotubes may reflect a cell defence against a subset of the mutant isoform, recognised as harmful misfolded proteins, by depositing them at the far end of the myotubes. The good incorporation of G53ins into filamentous structures and the induction of pathologically unremarkable phenotypes in differentiated myotubes might suggest that this β-TM mutant exerts a dominant effect via a functional defect rather than the formation of structural abnormalities.

The N202K mutant, linked to cap disease with the morphological features of fibre size variation and fibre type disproportion, in addition to cap structures [10], appeared mislocalised in the majority of the myoblasts. The N202K mutant did not readily integrate into actin filaments in the myotubes during differentiation but instead formed large aggregates in the cytoplasm. These accumulations were co-labeled with phalloidin, indicating that they were composed of polymerised actin. The tendency of the mutant TM predominantly to localise within cytoplasmic aggregates rather than incorporating into filamentous structures reduces the localisation of the N202K mutant within cytoplasmic thin filaments. The mutant TM may also affect the thin filament system via the disruption of the endogenous actin and the localisation of other sarcomeric components within cytoplasmic thin filaments. This was indicated by the marked reduction in the phalloidin labeling of actin filaments. Like the E41K mutant, the location of the N202K mutation in the outer region of the helixes is a good predictor of a protein interaction defect, [7], [10]. It is therefore likely that the disease is caused by *TPM2* mutations, which may differently affect both the functional and structural properties of β-TM by preventing it from having normal cellular dynamics.

Our expression studies in human cultured muscle cells demonstrated that the most striking abnormalities were nuclear and cytoplasmic accumulation and reduced incorporation into the filamentous structures. An enhanced capacity of K49del-β-TM_EGFP_, G53ins-β-TM_EGFP_, E122K-β-TM_EGFP_ and N202K-β-TM_EGFP_ to form insoluble protein filaments is supported by our histochemical findings in corresponding transfected myoblasts and differentiated myotubes, where fluorescent filamentous aggregates were discernible feature. To investigate the composition of the aggregates found in the transfected cells, we performed immunostaining with p62. It has been shown that p62 localises in aggregates common to several type of disease, acting as a polyubiquitinate-binding protein that plays an essential role in aggregate formation [26]. The findings in our immunostaining study indicated that p62 is expressed and selectively co-localised in the protein aggregates in transfected cells with the E41K-β-TM and E122K-β-TM mutants but not the WT-β-TM. This observation suggested that p62 may play a role in forming protein aggregates in myoblasts transfected with mutant TM, probably by linking misfolded or abnormal TM molecules together, in addition to a direct role for p62 in the discrimination between wild type and mutant TM.

Although the results may demonstrate the early stages of morphological changes linked to myopathy associated with *TPM2* mutations, our human tissue culture model did not reproduce the pathological features observed in patient muscle biopsies. This may not be surprising, because, in NM patients, rods were not identified in all muscle samples from multiple sites that were examined or in muscle biopsies taken at different time points [8], [36], [44], [45]. Similarly, an examination of four muscle samples from one individual with the M9R *TPM3* mutation indicated that the muscle pathology was not consistent in all muscle samples [37]. The different cellular environments are likely to be influenced differently by the underlying pathological processes, which result in a variable response to the mutant protein. This is partly due to the fact that, *in vivo*, the relative amounts of α-TM, β-TM and γ-TM expressed in a muscle fibre are characteristic of the muscle fibre type and dependent on the stage of development and other factors such as hormone status [46], [47]. Consequently, the appropriate composition of the TM isoforms is required for the appropriate function, dynamics and formation of TM dimers. *In vivo*, a change in the amino acid of TM is involved in the altered formation of α-helical coiled-coil heterodimers, TM composition and thin filaments, which in turn lead to alterations in sarcomeric composition [37]. Previously, we investigated the TM composition and the expression of TM isoforms in muscle specimens from patients carrying *TPM2* mutations, which indicated that *TPM2* mutations significantly alter the composition of the TM isoforms and modify the thin filament [48]. Other studies have also demonstrated that the Met9Arg *TPM3* mutation results in a preference for α/α dimer formation rather than α/β or β/β dimers likely to contribute to the severity of muscle weakness [37], [49], [50]. In addition, the sarcomere is a dynamic structure and the integration and exchange of new proteins occur continuously, as indicated by the constituent t_1/2_ life of sarcomeric proteins [51]. It is likely that mutated β-TMs disrupt the homeostasis of the sarcomere by affecting the t_1/2_ life of the protein. This, in turn, is likely to affect the assembly, dynamic turnover, filament stoichiometry and behaviour of individual sarcomeric proteins in the incorporation of newly synthesised myofilaments, which consequently lead to morphological changes. Thus, the variability in morphological features observed in patients' muscles and cellular models might be due to the different physiological contexts and variations in the ratio of mutant to wild-type TM. This is further supported by the differences between the fibre type composition in *in vivo* and cultured human myotubes. Our human cultures were mainly composed of the developmental myotube types (the embryonic and fetal) in contrast to the slow type 1 muscle fibres, where the β-TM is predominantly expressed *in vivo*.

Although skeletal muscle β-TM is highly conserved between humans and mice during evolution and differs in only one amino acid in position 66 (Fig. S1), β-TM mutants behaved differently in C2C12 compared with human culture muscle cells. This different behaviour of the β-TM mutants could be partly explained by differences in the expression pattern of endogenous β-TM in the two tissue culture models. The results obtained from immunofluorescence analysis indicated that β-TM is uniformly expressed in human myoblasts and its expression is increased in differentiated myotubes. In contrast, β-TM was not expressed in proliferating C2C12 myoblasts, but its expression was only detected in multinucleated elongated myotubes. These differences further support the hypothesis that changes in morphological features are not only influenced by the specific mutation, but could also be affected by the different cellular environments. Further, the appearance of rods in human muscle cells only when transfected with the K49del- and E122K-β-TM_EGFP_ constructs indicate that different mutations in the same protein can cause distinct morphological abnormalities.

## Conclusions

In conclusion, data from our human tissue culture model suggest the properties of β-TM mutants, which could be considered as the basis for the histological changes seen in muscle biopsies of patients and give us clues to the primary trigger for myopathy. Compared with C2C12, human cultured muscle cells, that are more environmentally closer to human skeletal muscle, more reliably mimic the disease conditions. In addition, we have demonstrated that the histopathological phenotypes associated with expression of different β-TM mutants are also influenced by the cellular environment, the degree of muscle maturation, and the temporal expression of the mutant protein.

## Supporting Information

Figure S1 **Alignment by** ***ClustalW*** **of residues within skeletal muscle β-TM of human and homologs of mouse showed that the residues are highly conserved.** Skeletal muscle β-TM differs in only one amino acid in position 66. Residues marked in red (asterisk): identical; green (two dots): similar.
(TIF)

Table S1 **Phenotypes of β-TM mutants expressed in human and C2C12 myoblasts and myotubes.**
(DOCX)

Table S2 **Transfected human and C2C12 myoblasts and myotubes classified into categories depending on β-TM_EGFP_ incorporation and the induced phenotypes.** Category 1: Whole cells with well-defined and organised filamentous structures. Category 2: Cells with poor integration of expressed β-TM_EGFP_ are divided into different subgroups depending on the induced phenotypes. Total number of the cells per construct that were investigated: 600 M: Myoblasts D: Differentiated cells.
(DOCX)

Diagram S1 **Distribution of different phenotypes of the expressed WT and mutant β-TM_EGFP_ constructs in human and C2C12 myoblasts and differentiated cells.** Phenotypes of β-TM mutants expressed in human (A) and C2C12 (B) cells differed before and after differentiation. The transfected myoblasts and myotubes were classified into categories depending on β-TM_EGFP_ incorporation and the induced phenotypes. The bars are subdivided, showing different phenotypes that were observed in the total number of 600 transfected myoblasts and myotubes per construct.M: Myoblasts, D: Differentiated cells.
(DOCX)
