# Supplementary figures and images for: Phenotypes of Myopathy-Related Beta-Tropomyosin Mutants in Human and Mouse Tissue Cultures
Source: PLoS One. 2013 Sep 10;8(9):e72396. doi: 10.1371/journal.pone.0072396 (PMC3769345; doi:10.1371/journal.pone.0072396)

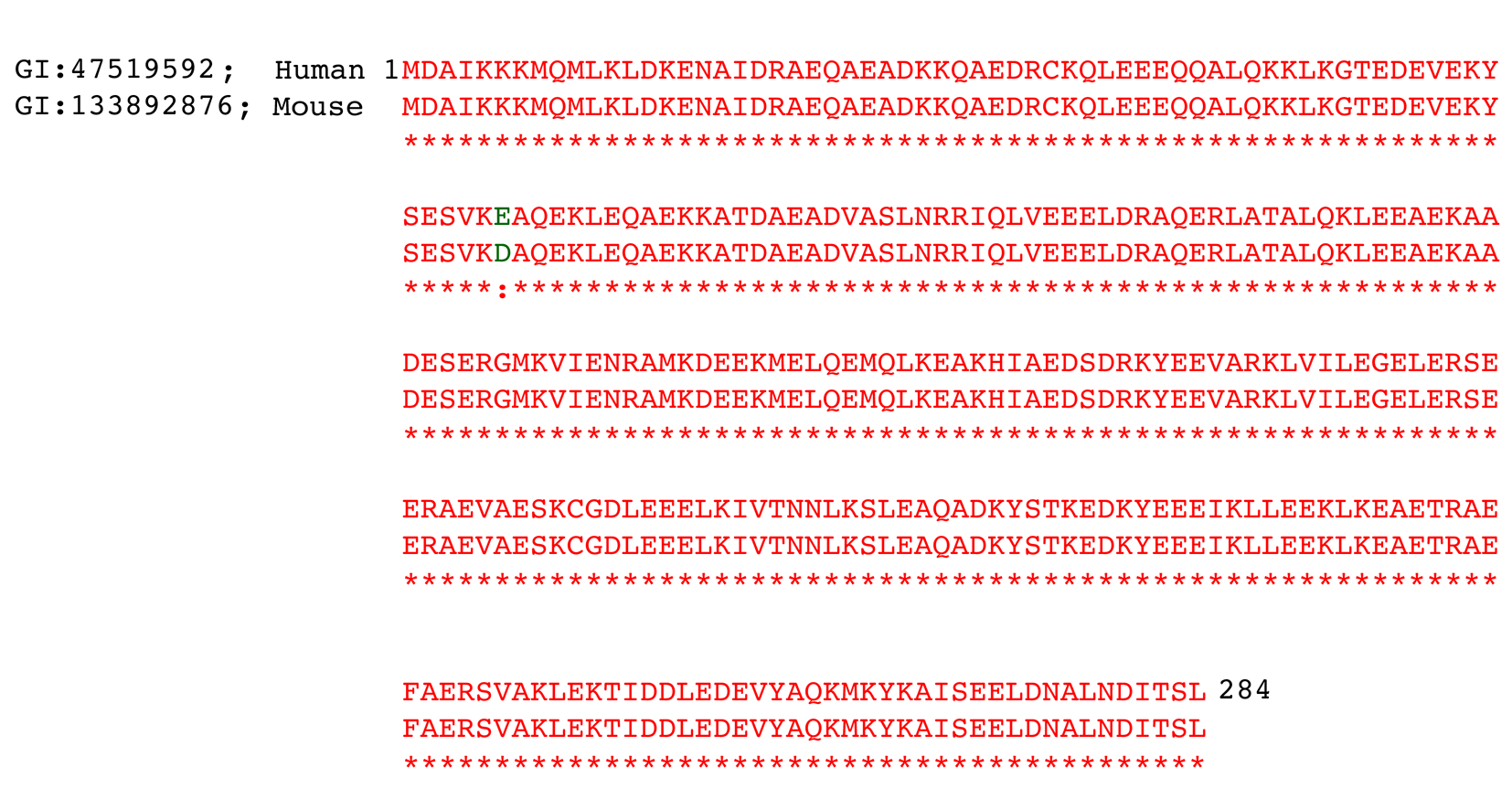

Supplement: Figure S1 — Alignment by ClustalW of residues within skeletal muscle β-TM of human and homologs of mouse showed that the residues are highly conserved. Skeletal muscle β-TM differs in only one amino acid in position 66. Residues marked in red (asterisk): identical; green (two dots): similar. (TIF) [file pone.0072396.s001.tif]
