# Supplementary material for: Phenotypes of Myopathy-Related Beta-Tropomyosin Mutants in Human and Mouse Tissue Cultures
Source: PLoS One. 2013 Sep 10;8(9):e72396. doi: 10.1371/journal.pone.0072396 (PMC3769345; doi:10.1371/journal.pone.0072396)
Supplement: Table S1 — Phenotypes of β-TM mutants expressed in human and C2C12 myoblasts and myotubes. (DOCX) [file pone.0072396.s002.docx]

**Table S1.**

| ***TPM2* mutations** | **Phenotypes in human myoblasts** | **Phenotypes in human differentiated cells** | **Phenotypes in mouse myoblasts** | **Phenotypes in mouse differentiated cells** |
| --- | --- | --- | --- | --- |
| **E41K** | Diffuse localisation, reduced cytoplasmic stress fibre incorporation | F-actin perinuclear aggregates | peripheral aggregates and diffuse cytoplasmic labeling of stress fibres | peripheral and perinuclear aggregates and reduced cytoplasmic labeling |
| **K49del** | Nuclear and cytoplasmic aggregates of endogenous actin, clouds in the nucleus and cytoplasm, cytoplasmic thickened filamentous, intranuclear rod-shaped structures, incorporation into stress fibres and filamentous lamellipodia | Cytoplasmic rod-shaped filaments, cytoplasmic aggregates and cloud-patterned, cytoplasmic thickened filamentous lamellipodia/actin structures | peripheral aggregates, diffuse labeling and nuclear and cytoplasmic aggregates | peripheral and perinuclear aggregates |
| **G53ins** | Endogenous actin aggregates and poor incorporation into filamentous structure of stress fibres | Integration into sarcomeric structure, diffuse cytoplasmic labeling at the far end of the myotubes | Mislocalisation, diffuse labeling, cytoplasmic aggregates and peripheral aggregates | peripheral aggregates |
| **E122K** | Cytoplasmic aggregates, intranuclear rod structures, cytoplasmic clouds with unorganised structure | Less well-defined phalloidin labeling and rod-like structures located at the far end of the myotubes | peripheral aggregates | peripheral and perinuclear aggregates |
| **N202K** | Small cytoplasmic aggregates, clouds around the nucleus and in the cytoplasm, diffuse cytoplasmic labeling | Thickened thin filaments and huge accumulation of filamentous actin | Diffuse cytoplasmic localisation, peripheral aggregates and thickened, ruffled cell surface | peripheral and perinuclear aggregates |
