# Supplementary material for: Phenotypes of Myopathy-Related Beta-Tropomyosin Mutants in Human and Mouse Tissue Cultures
Source: PLoS One. 2013 Sep 10;8(9):e72396. doi: 10.1371/journal.pone.0072396 (PMC3769345; doi:10.1371/journal.pone.0072396)
Supplement: Table S2 — Transfected human and C2C12 myoblasts and myotubes classified into categories depending on β-TMEGFP incorporation and the induced phenotypes. Category 1: Whole cells with well-defined and organised filamentous structures. Category 2: Cells with poor integration of expressed β-TMEGFP are divided into different subgroups depending on the induced phenotypes. Total number of the cells per construct that were investigated: 600 M: Myoblasts D: Differentiated cells. (DOCX) [file pone.0072396.s003.docx]

**Table S2.**

| **Human** **WT E41K K49del G53ins E122K N202K** | | | | | | | | | | | | |
| --- | --- | --- | --- | --- | --- | --- | --- | --- | --- | --- | --- | --- |
|  | M | D | M | D | M | D | M | D | M | D | M | D |
| Total number | 600 | 600 | 600 | 600 | 600 | 600 | 600 | 600 | 600 | 600 | 600 | 600 |
| **Category 1 %** | **80** | **70** | **25** | **20** | **20** | **21** | **15** | **13** | **9** | **10** | **22** | **11** |
| **Category 2 %** | **20** | **30** | **75** | **80** | **80** | **79** | **85** | **87** | **91** | **90** | **78** | **89** |
| Diffuse localisation | 80 | 70 | 90 | 10 | 2 | 0 | 25 | 95 | 3 | 77 | 20 | 10 |
| Cytoplasmic aggregates | 15 | 30 | 10 | 20 | 60 | 10 | 75 | 0 | 60 | 0 | 40 | 0 |
| Perinuclear aggregates | 0 | 0 | 0 | 70 | 0 | 0 | 0 | 0 | 0 | 0 | 0 | 0 |
| Nuclear aggregates | 0 | 0 | 0 | 0 | 7 | 0 | 0 | 0 | 0 | 0 | 0 | 0 |
| Cloud-patterned structures | 5 | 0 | 0 | 0 | 10 | 30 | 0 | 5 | 24 | 0 | 40 | 0 |
| Thickened filamentous structures | 0 | 0 | 0 | 0 | 7 | 20 | 0 | 0 | 0 | 0 | 0 | 10 |
| Intranuclear rod-shaped structures | 0 | 0 | 0 | 0 | 14 | 0 | 0 | 0 | 13 | 0 | 0 | 0 |
| Cytoplasmic rod-shaped structures | 0 | 0 | 0 | 0 | 0 | 40 | 0 | 0 | 0 | 23 | 0 | 0 |
| Huge accumulation of filamentous actin | 0 | 0 | 0 | 0 | 0 | 0 | 0 | 0 | 0 | 0 | 0 | 80 |
| **C2C12** |  |  |  |  |  |  |  |  |  |  |  |  |
| Total number | 600 | 600 | 600 | 600 | 600 | 600 | 600 | 600 | 600 | 600 | 600 | 600 |
| **Category 1 %** | **85** | **75** | **10** | **15** | **23** | **30** | **20** | **35** | **10** | **5** | **8** | **10** |
| **Category 2 %** | **5** | **25** | **90** | **85** | **77** | **70** | **80** | **65** | **90** | **95** | **92** | **90** |
| Peripheral aggregates | 0 | 0 | 60 | 45 | 45 | 39 | 35 | 88 | 70 | 50 | 35 | 50 |
| Perinuclear aggregates | 0 | 0 | 0 | 25 | 0 | 36 | 0 | 0 | 0 | 25 | 0 | 30 |
| Nuclear aggregates | 0 | 0 | 0 | 0 | 10 | 0 | 0 | 0 | 0 | 0 | 0 | 0 |
| Cytoplasmic aggregates | 5 | 4 | 0 | 0 | 25 | 0 | 50 | 0 | 0 | 0 | 0 | 0 |
| Diffuse staining | 95 | 96 | 40 | 30 | 20 | 25 | 15 | 11 | 30 | 20 | 15 | 20 |
| Thickened and ruffled cell surface | 0 | 0 | 0 | 0 | 0 | 0 | 0 | 0 | 0 | 0 | 50 | 0 |
