# Supplementary material for: Phenotypes of Myopathy-Related Beta-Tropomyosin Mutants in Human and Mouse Tissue Cultures
Source: PLoS One. 2013 Sep 10;8(9):e72396. doi: 10.1371/journal.pone.0072396 (PMC3769345; doi:10.1371/journal.pone.0072396)
Supplement: Diagram S1 — Distribution of different phenotypes of the expressed WT and mutant β-TMEGFP constructs in human and C2C12 myoblasts and differentiated cells. Phenotypes of β-TM mutants expressed in human (A) and C2C12 (B) cells differed before and after differentiation. The transfected myoblasts and myotubes were classified into categories depending on β-TMEGFP incorporation and the induced phenotypes. The bars are subdivided, showing different phenotypes that were observed in the total number of 600 transfected myoblasts and myotubes per construct.M: Myoblasts, D: Differentiated cells. (DOCX) [file pone.0072396.s004.docx]

**Diagram S1.**

A

B
